# Diversity of cell death signaling pathways in macrophages upon infection with modified vaccinia virus Ankara (MVA)

**DOI:** 10.1038/s41419-021-04286-3

**Published:** 2021-10-28

**Authors:** Lioba Klaas, Juliane Vier, Ian E. Gentle, Georg Häcker, Susanne Kirschnek

**Affiliations:** Faculty of Medicine, Institute of Medical Microbiology and Hygiene, Medical Center – University of Freiburg, Freiburg, Germany

**Keywords:** Phagocytes, Apoptosis, Necroptosis, Immune cell death, Viral infection

## Abstract

Regulated cell death frequently occurs upon infection by intracellular pathogens, and extent and regulation is often cell-type-specific. We aimed to identify the cell death-signaling pathways triggered in macrophages by infection with modified vaccinia virus Ankara (MVA), an attenuated strain of vaccinia virus used in vaccination. While most target cells seem to be protected by antiapoptotic proteins encoded in the MVA genome, macrophages die when infected with MVA. We targeted key signaling components of specific cell death-pathways and pattern recognition-pathways using genome editing and small molecule inhibitors in an in vitro murine macrophage differentiation model. Upon infection with MVA, we observed activation of mitochondrial and death-receptor-induced apoptosis-pathways as well as the necroptosis-pathway. Inhibition of individual pathways had a little protective effect but led to compensatory death through the other pathways. In the absence of mitochondrial apoptosis, autocrine/paracrine TNF-mediated apoptosis and, in the absence of caspase-activity, necroptosis occurred. TNF-induction depended on the signaling molecule STING, and MAVS and ZBP1 contributed to MVA-induced apoptosis. The mode of cell death had a substantial impact on the cytokine response of infected cells, indicating that the immunogenicity of a virus may depend not only on its PAMPs but also on its ability to modulate individual modalities of cell death. These findings provide insights into the diversity of cell death-pathways that an infection can trigger in professional immune cells and advance our understanding of the intracellular mechanisms that govern the immune response to a virus.

## Introduction

The modified vaccinia virus Ankara (MVA) is a member of the poxviruses, a group of large dsDNA-viruses that includes the agent of smallpox and viruses used for vaccination such as vaccinia virus (VACV). MVA is a VACV-derivative that has been further attenuated to improve the safety of anti-smallpox vaccines and that has lost about 10% of the original genome in the process. It maintains high immunogenicity and replicates only in avian cells but is replication-incompetent in human or mouse cells [1]. MVA is under investigation as a vaccination vector against various infectious diseases and for cancer immunotherapy [2].

Cell death plays an important role in the host response to viral infection. Apoptosis is considered a mechanism of the host cell to limit viral replication, and many viruses are known to have evolved proteins to interfere with the host cell death machinery [3–6]. In epithelial cells, MVA inhibits host cell death [4].

Naturally, poxviruses probably mainly infect epithelial cells of the skin, but will also come into contact with macrophages and various other innate immune cells [7], especially as a vaccination vector. During systemic infection of mice, macrophages and dendritic cells (DC) were the main targets of MVA [8]. Predominant infection of MHCII-expressing antigen-presenting cells was observed in several animal species using various administration routes [9]. In epithelial cells, viral components inhibit the host cell’s attempt to undergo apoptosis. In myeloid cells, the situation seems different. Infection of human DC with VACV and MVA in vitro resulted in abortive infection accompanied by apoptotic cell death [10]. Infection of bovine DC in vitro with MVA led to caspase-like activity and reduction of T cell-stimulatory molecules [11]. In murine bone marrow-derived DC, MVA induced both cell death and autophagy, important for cross-priming of OVA-specific cytotoxic T cells [12]. In mouse macrophages, MVA-infection has a substantial proapoptotic potential [13]. Although apoptosis is the longest known cell death response to viral infection [14], it is becoming apparent that other forms of regulated cell death, especially necroptosis, may play a role [15]. The mode of cell death and its link to virus-induced inflammatory pathways likely affects the immune response to the virus.

Here we investigated the cell death-pathways activated in mouse macrophages upon infection with MVA. We targeted components of specific cell death-pathways using genome editing and small molecule inhibitors in an in vitro murine macrophage differentiation model. Our results suggest that autocrine TNF-signaling is an important factor in both apoptosis and necroptosis-induction and that cytosolic DNA recognition, especially via the STING-pathway, is relevant for the regulation of both the cell death response and the innate immune response to the viral infection.

## Results

### MVA-infection of macrophages can activate both main apoptosis-pathways as well as necroptosis

We used an in vitro model of macrophage generation by differentiation from “conditionally immortalized” murine bone marrow precursors [16]. The differentiated cells closely resemble inflammatory monocyte-derived macrophages [17]. This model permits in vitro generation of basically unlimited numbers of terminally differentiated cells and easy genetic modification. Typical morphology and surface marker profiles are shown in Fig. S1a, b.

**Fig. 1:**
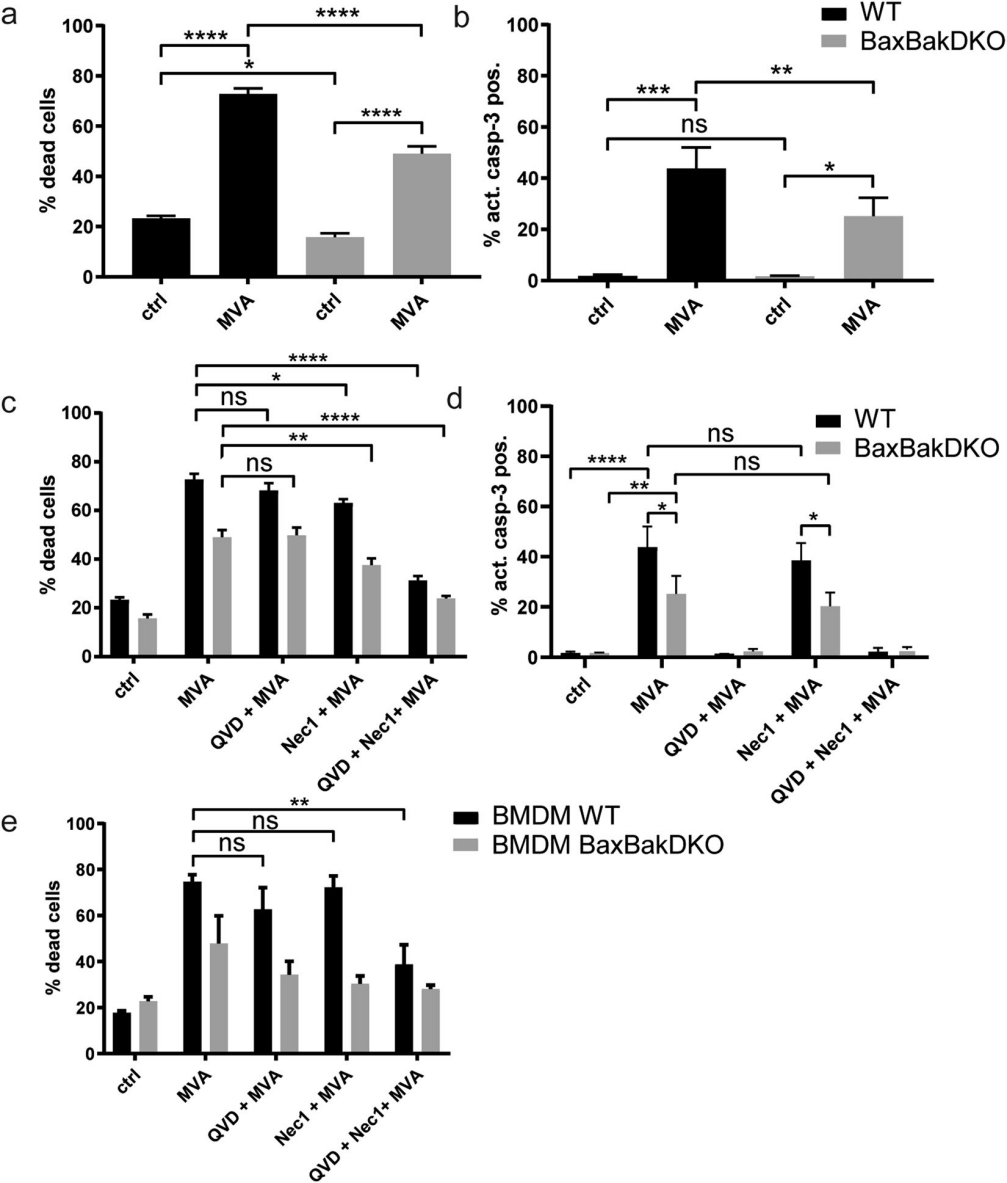
MVA activates apoptosis and necroptosis-pathways in macrophages. **a**–**d** D7 differentiated wt (black bars) or Bax/Bak-DKO (gray bars) Hoxb8 macrophages were infected with MVA at an MOI of 2 and harvested after 22 h by accutase treatment followed by annexin/PI staining (**a**, **c**) or active caspase-3 staining (**b**, **d**) and flow cytometry analysis. **e** Primary macrophages (differentiated from murine bone marrow in the presence of M-CSF, BMDM) were infected with MVA at an MOI of 2 and harvested after 22 h by accutase treatment followed by annexin V/PI staining and flow cytometry. The rate of dead cells was determined as a percentage of annexin V/PI-positive cells (defined as cells positive for annexin V or PI or both). Inhibitors were used at the following concentrations where indicated: QVD, 20 µM; Necrostatin-1 (Nec-1), 10 µM. Shown are means/SEM of *n* = 6 (**a**, **c**) or *n* = 5 (**b**, **d**) experiments. *p < 0.05; **p < 0.01; ***p < 0.001; ****p < 0.0001; ns nonsignificant (*p* ≥ 0.05).

It has been reported before that infection with MVA or VACV causes cell death in macrophages [13, 18]. Hoxb8 wt macrophages were infected with MVA (MOI 2), and cell death was measured by analysis of annexin V-binding and plasma membrane integrity. We observed massive cell death with about 70% of annexin V/PI-positive cells 22 h post infection (Figs. 1a and S2a). Around 70% of dead cells (50% of all infected cells) had active caspase-3, indicating that many of the cells died by apoptosis (Figs. 1b and S2b). We first addressed the contribution of the mitochondrial apoptosis-pathway. Bax/Bak-double-deficient Hoxb8 cells (lacking a functional mitochondrial apoptosis-pathway) were generated [19]. We observed a small decrease in the rate of annexin V/PI-positive cells, and a more prominent decrease in caspase-3 positivity (Fig. 1a, b). Thus, MVA-infection induced substantial apoptosis through both mitochondrial and non-mitochondrial pathways. In addition, non-apoptotic cell death (loss of membrane integrity in the absence of caspase-activation) occurred in the absence of Bax/Bak. This suggested activation of the intrinsic mitochondrial apoptosis-pathway, extrinsic apoptosis, and additional non-apoptotic cell death pathways.

We used the pan-caspase inhibitor QVD-OPh (QVD) to inhibit apoptosis and the RIPK1-inhibitor necrostatin-1 (Nec-1) to block necroptosis. QVD and/or Nec-1 treatment alone did not alter macrophage viability (Fig. S3a, b). QVD had no effect on cell death rates in wt or Bax/Bak-double-deficient MVA-infected macrophages (Fig. 1c). Nec-1 slightly decreased the rates of annexin V/PI-positive cells in MVA-infected wt or Bax/Bak-double-deficient cells and had slight effects on caspase-3 activation (Fig. 1d). Simultaneous inhibition of caspases and RIPK1 however almost completely blocked MVA-induced macrophage death (Fig. 1c). This indicated that both apoptosis and necroptosis-pathways can be activated in macrophages by MVA-infection and suggests that blockage of one pathway causes a shift to the other. Only in the absence of both caspase-activity and RIPK1-activity, MVA-induced cell death was efficiently blocked. Results were confirmed in primary myeloid cells (Fig. 1e). In bone marrow-derived macrophages (BMDM) from wt mice, MVA-treatment similarly induced cell death that was blocked by the combination of QVD/Nec-1. In MVA-infected Bax/BakDKO BMDM, Nec-1 had a strong cell death-inhibitory effect comparable to the combination with QVD. This indicates a switch to mainly RIPK1-dependent death when mitochondrial apoptosis-signaling was blocked. In human peripheral blood-derived monocytes, MVA-infection induced cell death comparable to murine cells (Fig. S4). The combination of QVD and Nec-1 efficiently blocked MVA-induced cell death, although protection by QVD alone was stronger than in the mouse cells. Nec-1 alone slightly reduced the percentage of annexin V/PI and active caspase-3-positive MVA-infected cells (Fig. S4a, b). The stronger effect of QVD on overall cell death and Nec-1 on caspase-3-activity indicates that here the balance may shift more towards apoptosis.

**Fig. 2:**
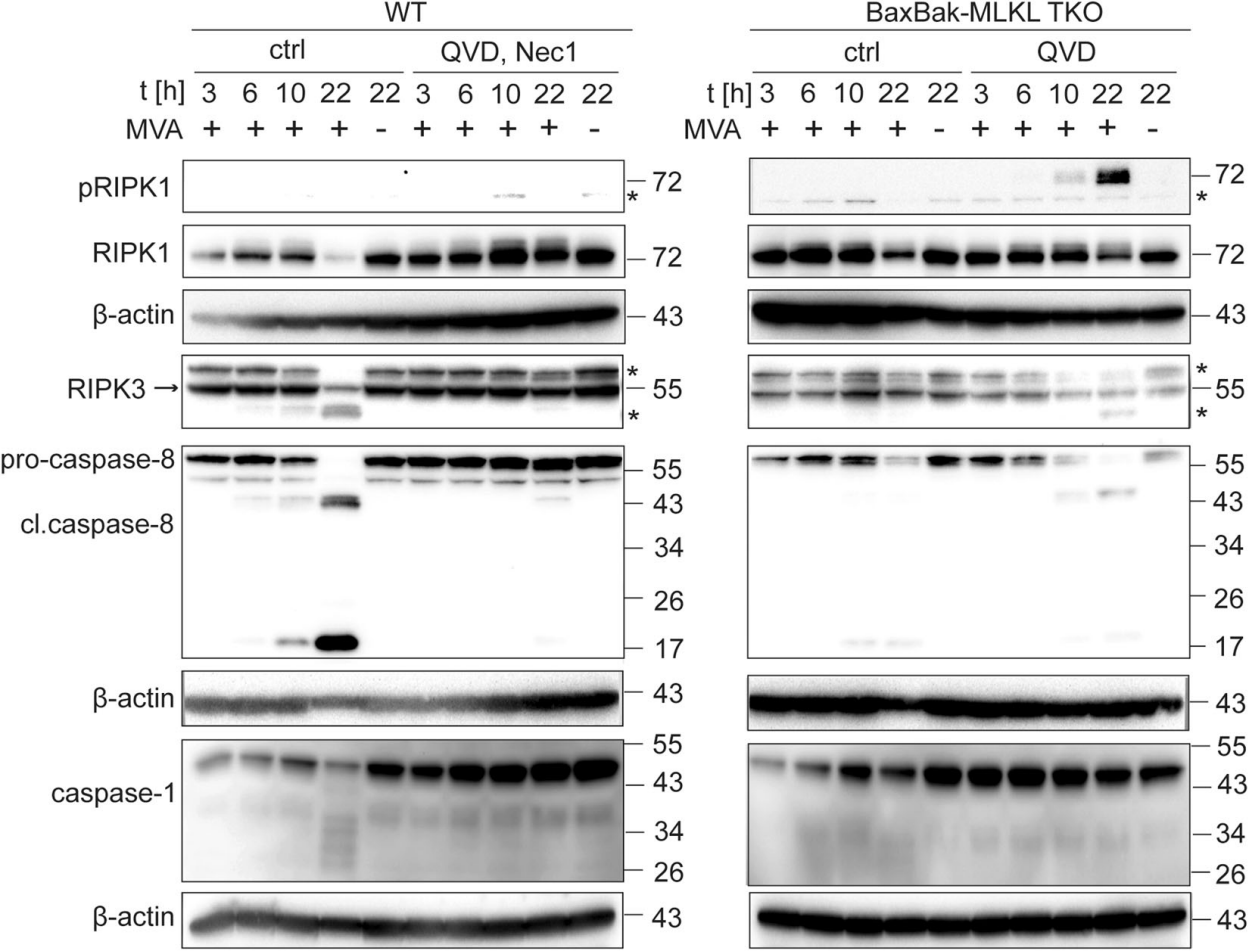
Western blot analysis of cell death-associated signaling proteins upon MVA-infection in macrophages. D7 differentiated wt (left) or Bax/Bak/MLKL TKO (right) Hoxb8 macrophages were infected with MVA at an MOI of 2 and harvested at the indicated time points by accutase treatment. Cells were lysed in Laemmli sample buffer and samples were boiled at 95 °C for 5 min. Samples were subjected to SDS-PAGE on a 4–20% TRIS-glycine gel, transferred onto PVDF membranes, and probed with the antibodies indicated. Inhibitors were used at the following concentrations where indicated: QVD, 20 µM; Necrostatin-1 (Nec-1), 10 µM. β-actin served as a loading control. *unspecific signal. Blots are representative of three independent experiments.

We monitored the cleavage of apoptotic caspases by Western blot. In wt cells, MVA-infection induced strong cleavage of caspase-8, caspase-9, and caspase-3 (Figs. 2 and S5a–d). QVD together with Nec-1 efficiently prevented caspase-8 processing. The caspase-8-targets RIPK1 and RIPK3 were cleaved. In Bax/Bak-double-deficient cells, caspase-9-cleavage was blocked as expected, and caspase-3-cleavage was reduced (Fig. S5a–d). In these cells and in Bax/Bak/MLKL-triple-deficient (TKO) cells (resistant to mitochondrial apoptosis and to MLKL-mediated loss of membrane integrity) we observed reduced and delayed caspase-8-activation (Figs. 2 and S5a–d). This suggests that this cleavage is in part secondary to mitochondrial apoptosis, most likely through caspase-3. Similar caspase-cleavage profiles were seen in primary murine macrophages (Fig. S5d). We also observed phosphorylation of RIPK1 in the presence of QVD, supporting the concept that caspase-inhibition shifts the response towards necroptosis (Fig. 2). A slight smear of possible cleavage/degradation products of caspase-1 was observed in wt but not Bax/Bak/MLKL TKO cells, but no distinct cleavage product was seen (Fig. 2). To test for activation of necroptosis, we analyzed the phosphorylation status of MLKL. MVA-infection resulted in a time-dependent increase in MLKL phosphorylation in Hoxb8 and primary macrophages (Fig. S5b, e). Phosphorylated MLKL was hardly detectable in MVA-infected primary Bax/BakDKO cells unless caspases were inhibited. This indicates that Nec-1-mediated protection in Bax/Bak-deficient primary cells may be mainly mediated by limiting RIPK1-dependent caspase-8/3-signaling. Caspase-inhibition by QVD however led to enhanced MLKL phosphorylation comparable in both WT and Bax/BakDKO cells. This suggests that QVD drives a shift to necroptotic signaling in this context (Fig. S5b, e).

Cell death regulation in macrophages upon MVA-infection seems to be peculiar to this cell type. In epithelial cells, strong antiapoptotic effects have been reported for infection with MVA and the vaccinia virus [3, 4]. Analysis of the caspase-activation profile in MVA-infected HeLa epithelial cells revealed limited cleavage of caspase-8 and -9, but no detectable caspase-3-cleavage (Fig. S6a). Consistent with this and earlier reports, cell death levels in MVA-infected HeLa cells were only slightly elevated (Fig. S6b, c).

### Upstream signaling pathways involved in MVA-induced cell death

**Fig. 3:**
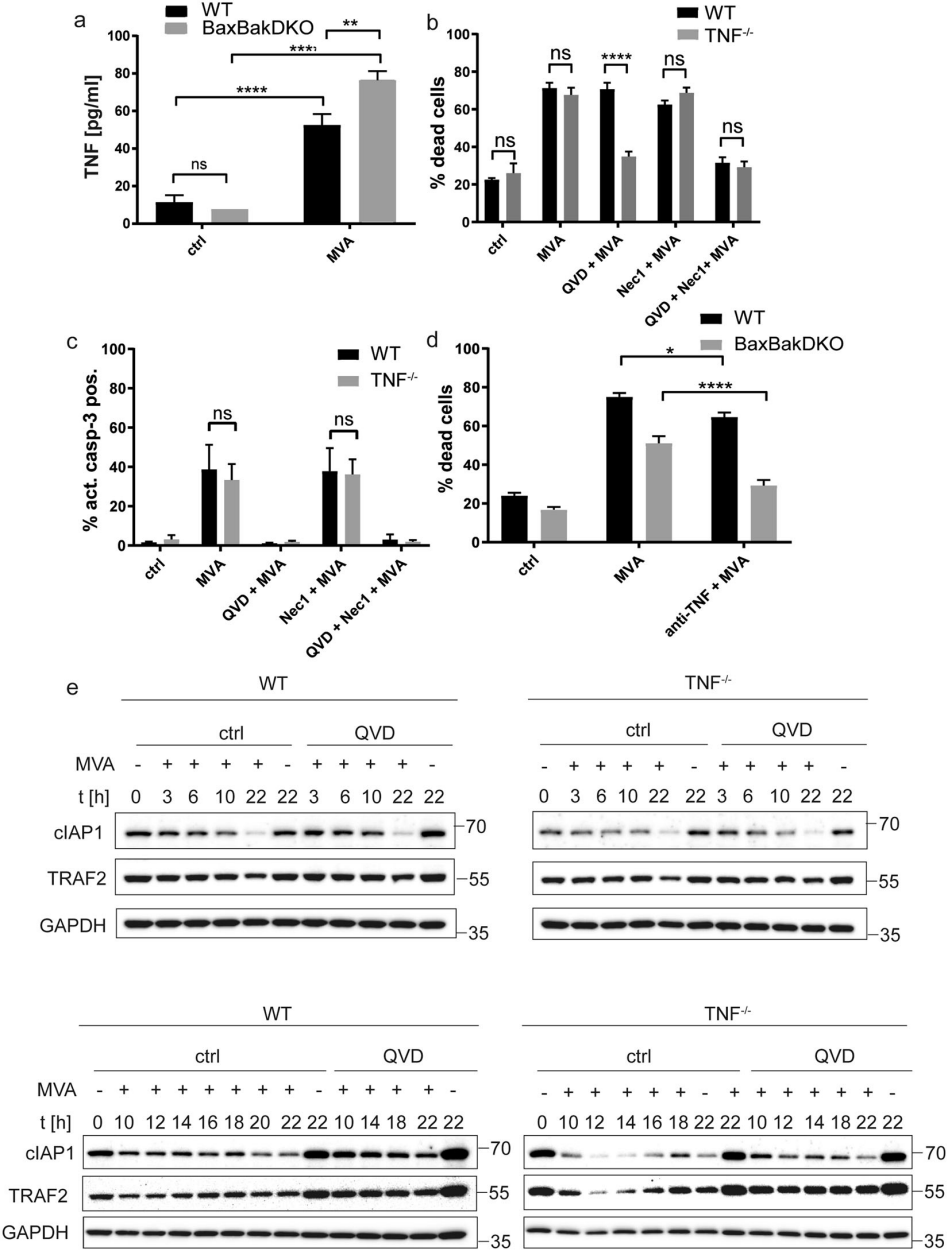
Involvement of TNF-signaling in MVA-induced macrophage cell death. **a**, **b** D7 differentiated wt (black bars) or Bax/Bak-DKO (gray bars) Hoxb8 macrophages were infected with MVA at a MOI of 2 and harvested after 22 h by accutase treatment followed by annexin/PI staining (**a**) or active caspase-3 staining (**b**) and flow cytometry analysis. Inhibitors were used at the following concentrations where indicated: QVD, 20 µM; Nec-1, 10 µM. **c** Supernatants of d7 differentiated wt (black bars) or Bax/Bak-DKO (gray bars) Hoxb8 macrophages infected with MVA at an MOI of 2 for 22 h were analysed for secreted TNF by ELISA. **d** D7 differentiated wt (black bars) or Bax/Bak-DKO (gray bars) Hoxb8 macrophages were infected with MVA at a MOI of 2 and harvested at the indicated time points by accutase treatment. Where indicated, samples were concomitantly treated with anti-TNF antibody (1:1000). Cells were analysed for cell death by annexin V/PI staining followed by flow cytometry. The rate of dead cells was determined as a percentage of annexin/PI-positive cells (cells positive for annexin V or PI or both). **e** D7 differentiated wt or TNF^−/−^ Hoxb8 macrophages were infected with MVA at an MOI of 2 and harvested at the indicated time points by accutase treatment. Cells were lysed in Laemmli sample buffer and samples were boiled at 95 °C for 5 min. Samples were subjected to SDS-PAGE on 4–20% TRIS-glycine gels, transferred onto nitrocellulose membranes, and probed with the antibodies indicated. The pan-caspase inhibitor QVD was used at a concentration of 20 µM where indicated. GAPDH served as a loading control. Blots are representative of at least two independent experiments. Shown are means/SEM of *n* = 4 (**a**, **b**, **d**) or *n* = 6 (**c**) independent experiments. *p < 0.05; **p < 0.01; ***p < 0.001; ****p < 0.0001; ns nonsignificant (*p* ≥ 0.05).

A multitude of cytokines have been shown to be secreted by MVA-infected macrophages and may be modulated by cell death-signaling. Conversely, cytokine production by myeloid cells induced by MVA-infection may have an impact on apoptotic and necroptotic cell death-induction in an autocrine/paracrine manner. One likely candidate for contribution to apoptotic and necroptotic cell death is TNF, which is induced in macrophages by various viral stimuli. The amount of TNF secreted upon MVA-stimulation (around 50–150 pg/ml, Figs. 3a, 5e) seemed however too low to explain the cell death observed. In uninfected macrophages treated with recombinant murine TNF, no significant cell death occurred even with much higher concentrations (Fig. S7). This does not exclude a role for TNF however: TNF may act in its membrane-inserted form, or infection may sensitize cells to TNF-signaling. The requirement for TNF was tested using TNF-deficient macrophages. TNF deficiency alone did not alter cell death susceptibility to MVA-infection, but QVD significantly blocked MVA-induced cell death in the absence of TNF (Fig. 3b, c); Nec-1 had no effect. We further tested the contribution of TNF-signaling using a TNF-blocking antibody. Cell death in wt cells was only slightly affected by neutralization of TNF in line with results of TNF-deficient cells. In contrast, anti-TNF-antibodies significantly inhibited cell death-induction by MVA-infection in Bax/Bak-double-deficient cells (Fig. 3d). Remarkably, protein levels of TNF-receptor complex components cIAP1/TRAF2 declined over time largely independent of TNF (Fig. 3e). Such a depletion of cIAP1/TRAF2 could facilitate caspase-8-dependent apoptosis or necroptosis and may explain the strong sensitization to TNF-dependent cell death despite low detectable amounts of TNF. Bax/Bak-deficiency slightly reduced the loss of both proteins (Fig S5c). This suggests a minor contribution of mitochondrial apoptosis-signaling. Taken together, this argued on the one hand for a switch to strongly TNF-dependent necroptosis under caspase blockade, on the other hand for ongoing TNF-independent, caspase-dependent death when necroptosis was blocked. TNF appeared to induce either apoptosis or when caspases were inhibited, necroptosis.

**Fig. 4:**
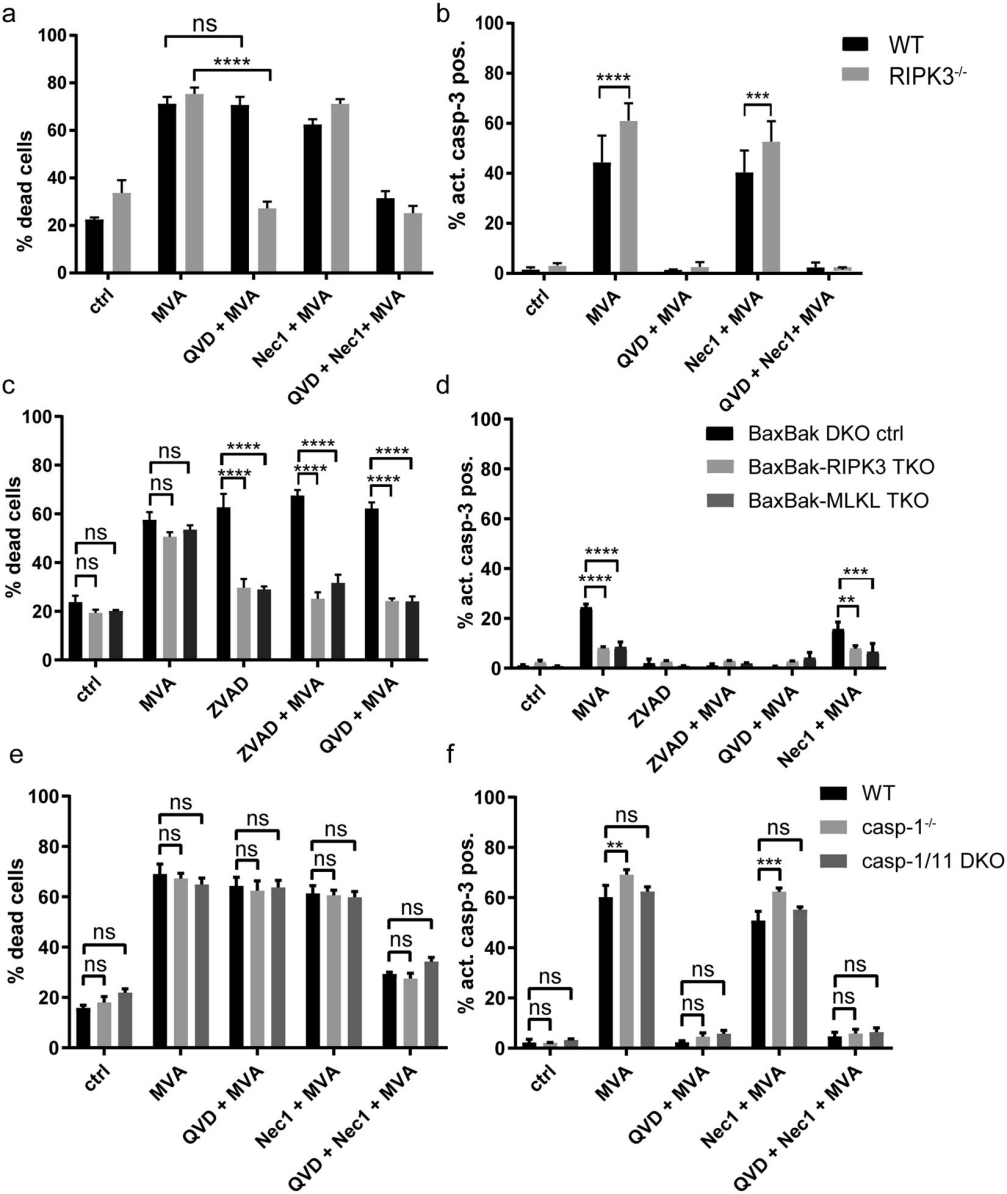
Specific contribution of the necroptosis-pathway to MVA-induced cell death. **a**, **b** D7 differentiated wt (black bars) or RIPK3^−/−^ Hoxb8 macrophages (gray bars) were infected with MVA at an MOI of 2 and harvested after 22 h by accutase treatment followed by annexin/PI staining (**a**) or active caspase-3 staining (**b**) and flow cytometry analysis. **c**, **d** Bax/Bak-RIPK3-TKO (light gray bars) or Bax/Bak-MLKL TKO macrophages (dark gray bars), genetically modified by CRISPR/Cas9, were infected with MVA at an MOI of 2 and harvested after 22 h by accutase treatment. Cells were subjected to annexin/PI staining (**c**) or active caspase-3 staining (**d**) followed by flow cytometry analysis. Bax/Bak-DKO ctrl cells expressing an irrelevant gRNA directed against EGFP served as control (black bars). **e**, **f** D7 differentiated wt (black bars), Caspase-1^−/−^ (gray bars), or Caspase-1/11 DKO Hoxb8 macrophages were infected with MVA at an MOI of 2 and harvested after 22 h by accutase treatment followed by annexin/PI staining (**e**) or active caspase-3 staining (**f**) and flow cytometry analysis. Inhibitors were used where indicated at the following concentrations: QVD, 20 µM; ZVAD, 50 µM; Nec-1, 10 µM. Shown are means/SEM of *n* = 4 (**a**, **b**) or *n* = 3 (**c**–**e**) independent experiments. *p < 0.05; **p < 0.01; ***p < 0.001; ****p < 0.0001; ns nonsignificant (*p* ≥ 0.05).

RIPK3 is an essential mediator of necroptosis downstream of TNF-signaling and RIPK1-activity. Interestingly, RIPK3-deficient cells behaved like TNF-KO cells: upon MVA-infection, RIPK3-deficiency conferred no protection (Fig. 4a). There was a small increase of caspase-3-positive cells in the absence of RIPK3 arguing for sensitization to apoptosis (Fig. 4b). The presence of QVD blocked MVA-induced death in RIPK3-deficient cells efficiently (Fig. 4a). This demonstrated that the caspase-independent component of MVA-mediated cell death-signaling was controlled by TNF-mediated, RIPK3-dependent necroptosis.

### Specific contribution of the necroptosis-pathway to MVA-induced cell death

We deleted either RIPK3 or the downstream necroptosis mediator MLKL in Bax/Bak-double-deficient cells (Fig. S8). This background was chosen to separate intrinsic from extrinsic apoptosis. Bax/Bak-double-deficient cells expressing Cas9 and an irrelevant gRNA served as controls. Differentiation of the cells was unaffected (Fig. S1). Deletion of either RIPK3 or MLKL alone had no effect on overall cell death. However, under conditions of caspase-inhibition (ZVAD-fmk or QVD), the absence of either RIPK3 or MLKL abrogated MVA-induced cell death (Fig. 4c, d). This indicated that MVA-induced caspase-independent cell death depends on a functional RIPK3-MLKL-mediated necroptosis pathway.

Pyroptosis, a lytic form of inflammasome-mediated, caspase-1/11-dependent cell death, can contribute to inflammatory cell death induced by intracellular pathogens in various cell types [20]. To test for contribution of caspases-1 or -11, caspase-1-deficient and caspase-1/11 double-deficient Hoxb8 macrophages were infected with MVA. There was no difference in cell death in either cell line compared to wt cells (Fig. 4e, f). Neither single treatment with QVD nor Nec-1 protected the cells (Fig. 4e, f), making the involvement of caspase-1 and/or -11 in the regulation of MVA-induced cell death unlikely.

### Modulation of the cytokine response of MVA-infected macrophages by distinct cell death-pathways

**Fig. 5:**
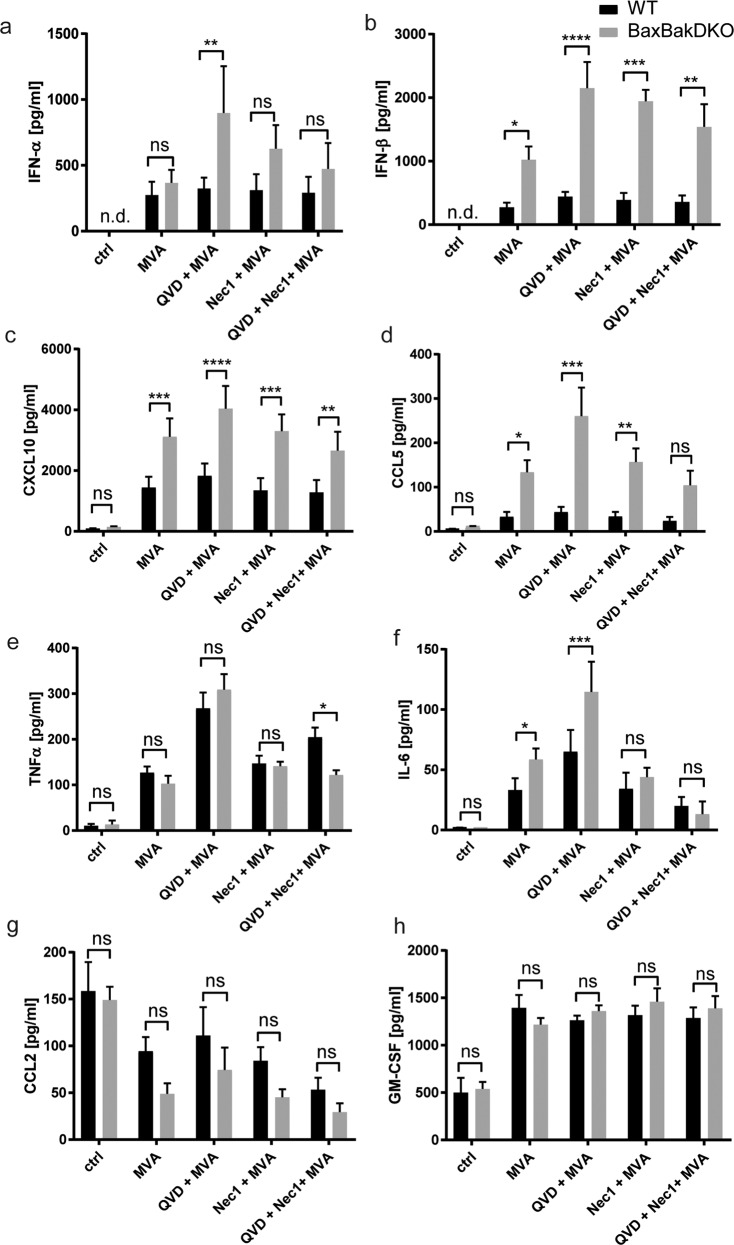
Modulation of the cytokine response of MVA-infected macrophages by distinct cell death-pathways. **a**–**h** Supernatants of d7 differentiated wt (black bars) or Bax/Bak-DKO (gray bars) Hoxb8 macrophages infected with MVA at a MOI of 2 were collected after 22 h. Some samples were treated with inhibitors QVD (20 µM) and Nec-1 (10 µM) alone or in combination as indicated. Samples were analysed for the cytokines indicated by a bead-based assay (Legendplex, antivirus response panel, Biolegend). Shown are the means/SEM of *n* = 3 independent experiments. *p < 0.05; **p < 0.01, ***p < 0.001, ****p < 0.0001; ns nonsignificant (*p* ≥ 0.05).

An important part of the innate immune response is the production of cytokines and chemokines upon microbial contact, and cell death-pathways can interfere with cytokine production. We tested how the MVA-induced cytokine/chemokine response in macrophages was altered upon inhibition of distinct cell death pathways. As expected, MVA-infection strongly induced secretion of type 1 interferons (IFN-I) and the IFN-stimulated gene products CXCL10 and CCL5 in macrophages, as well as IL-6, TNF, and GM-CSF (Fig. 5). When the mitochondrial apoptosis pathway was blocked by Bax/Bak-deletion, secretion of IFNβ, CXCL10, CCL5, and IL-6 was strongly enhanced. In contrast, TNF and GM-SCF-secretion was not significantly affected. Pan-caspase-inhibition during MVA-infection similarly led to increased TNF and IL-6-secretion, whereas the IFN-I response was induced by additional QVD treatment in Bax/Bak-double-deficient, but not wt cells. Additional inhibition of necroptosis using Nec-1 abrogated the QVD-induced TNF and IL-6-induction. No IL1β, CXCL1, IL10, or IL12-secretion was detectable (not shown). Taken together, the results indicated that caspase activation contributes to the silencing of IFN-I and IL-6 via both the mitochondrial and the extrinsic apoptosis-pathway, and modulates the TNF response exclusively through the extrinsic apoptosis pathway.

TNF deficiency had no direct impact on most other cytokines investigated, except IL-6, which was reduced in the absence of TNF (Fig. S9a–h). Strikingly, suppression of constitutive CCL2 was observed during MVA-infection, especially in TNF-deficient cells (Figs. 5g and S9g). RIPK3-deficiency reduced IL-6-secretion in MVA-stimulated cells, similar to TNF-deficiency (Fig. S9f), and somewhat reduced IFNα and CXCL10-secretion (Fig. S9a, c). Moreover, the constitutive secretion of CCL2 strongly depended on RIPK3 (Fig. S9g). This indicates the presence of constitutive RIPK3-activity in macrophages, which is modulated by MVA.

### Contribution of DNA recognition pathways to cell death-signaling in MVA-infected macrophages

Nucleic acid recognition pathways play an important role in recognizing viruses and subsequent induction of antiviral responses and can modulate cell death-signaling upon viral infection. Zbp1/DAI has been reported to trigger cell death during infection with murine cytomegalovirus (MCMV) [21], influenza virus [22], or VACV [6]. STING-dependent signaling plays a role in virulence [23, 24] and necroptosis-induction [25] during poxvirus infection. MAVS has been shown to contribute to apoptosis-induction by MVA in epithelial cells [8]. Innate immune sensing of MVA and production of IFNβ and CXCL10 seemed dependent on MDA-5/MAVS in THP-1 cells [26].

**Fig. 6:**
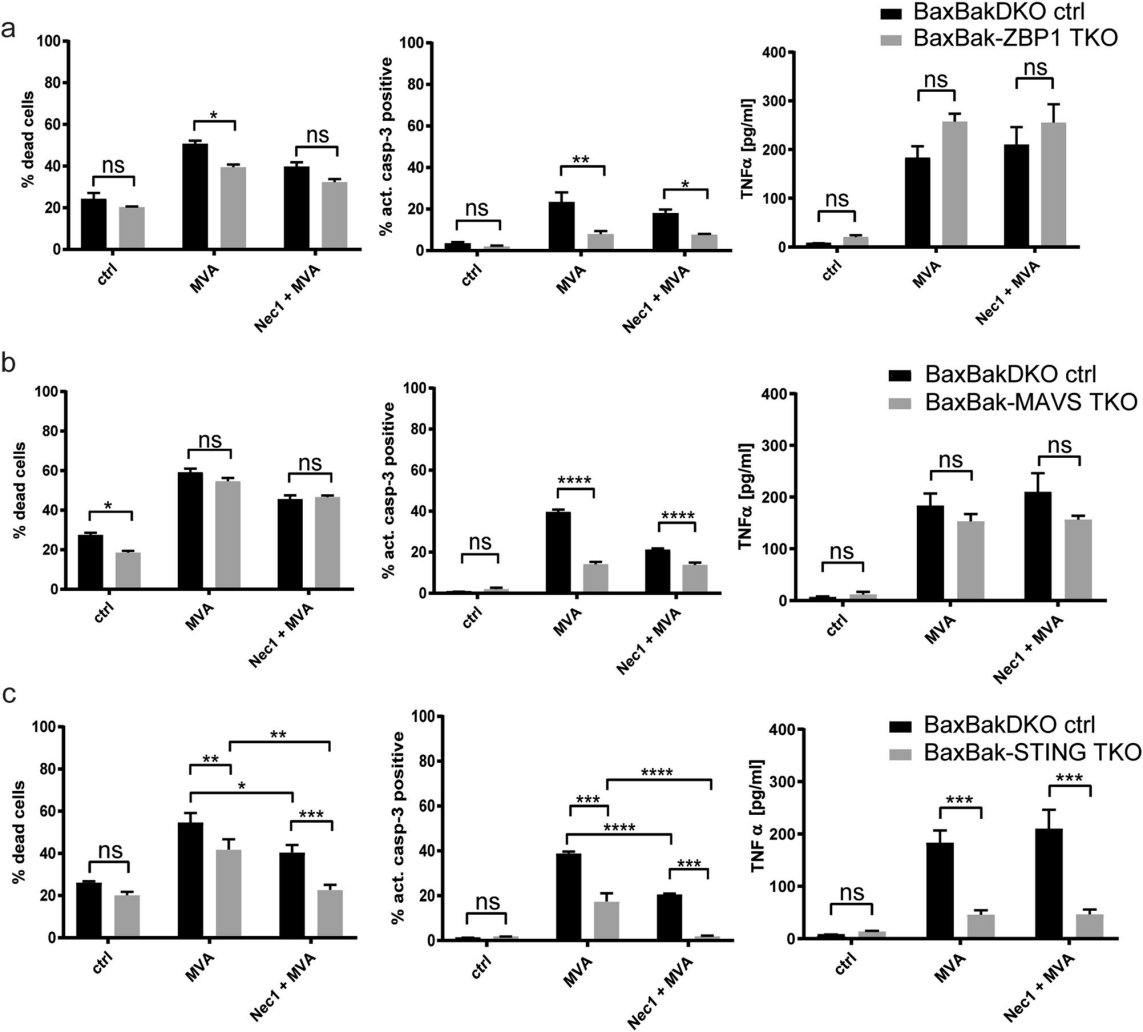
Contribution of DNA recognition pathways to cell death-signaling in MVA-infected macrophages. **a**–**c** Bax/Bak-ZBP1 TKO (**a**, gray bars), or Bax/Bak-MAVS TKO (**b**, gray bars), or Bax/Bak-STING TKO cells (genetically modified by CRISPR/Cas) were infected with MVA at an MOI of 2 and cells and supernatants were harvested after 22 h. Cells were stained for annexin/PI (left panels) or active caspase-3 (middle panels) followed by flow cytometry analysis. Supernatants were analysed for TNF-secretion by a bead-based assay (right panels). Bax/Bak-DKO ctrl cells expressing an irrelevant gRNA directed against EGFP served as control (black bars). The inhibitor Nec-1 (10 µM) was added were indicated. Shown are the means/SEM of *n* = 3 independent experiments. *p < 0.05; **p < 0.01, ***p < 0.001, ****p < 0.0001; ns nonsignificant (*p* ≥ 0.05).

We deleted several components of DNA recognition-pathways on a Bax/Bak-deficient background to prevent interference by the mitochondrial apoptosis pathway. Loss of DAI/Zbp1 conferred slight protection against MVA-induced cell death but strongly decreased caspase-3 activation. This indicated that DAI/Zbp1-signaling was involved in mitochondria-independent apoptosis-induction but did probably not contribute to necroptosis (Fig. 6a). MAVS-deficiency had no effect on overall cell death, but reduced caspase-3 activation (Fig. 6b). STING-deficiency however substantially reduced caspase-3 activation upon MVA-infection. Nec-1 treatment, which reduced cell death in Bax/Bak-deficient cells, was able to block both MVA-induced overall cell death and to abrogate caspase-3 activation in Bax/Bak-STING-deficient cells (Fig. 6c). This clear effect of STING on cell death regulation correlated with its impact on TNF-production: whereas DAI/Zbp1-deficiency led to increased TNF-production upon MVA-infection and MAVS-deficiency had no effect, loss of STING substantially reduced TNF-secretion (Fig. 6c). The residual caspase-8-cleavage in MVA-infected Bax/BakDKO cells proved to be strongly STING/TNF-dependent. This was reflected by the strong reduction of caspase-8-cleavage in STING-deficient Bax/BakDKO cells and almost complete lack of cleavage when TNF was blocked by neutralizing anti-TNF-antibodies (Fig. S10a). Similarly, MLKL phosphorylation was prevented by TNF neutralization and reduced by STING deficiency (Fig. S10b). Altogether, the data indicated that STING-deficiency prevented MVA-induced Bax/Bak-independent, RIPK1-independent apoptosis, whereas RIPK3-dependent necroptosis was partially STING-independent. This further suggests that STING-mediated TNF-secretion is largely responsible for the mitochondria-independent apoptosis-signaling.

### Influence of DNA recognition-pathways on macrophage activation

**Fig. 7:**
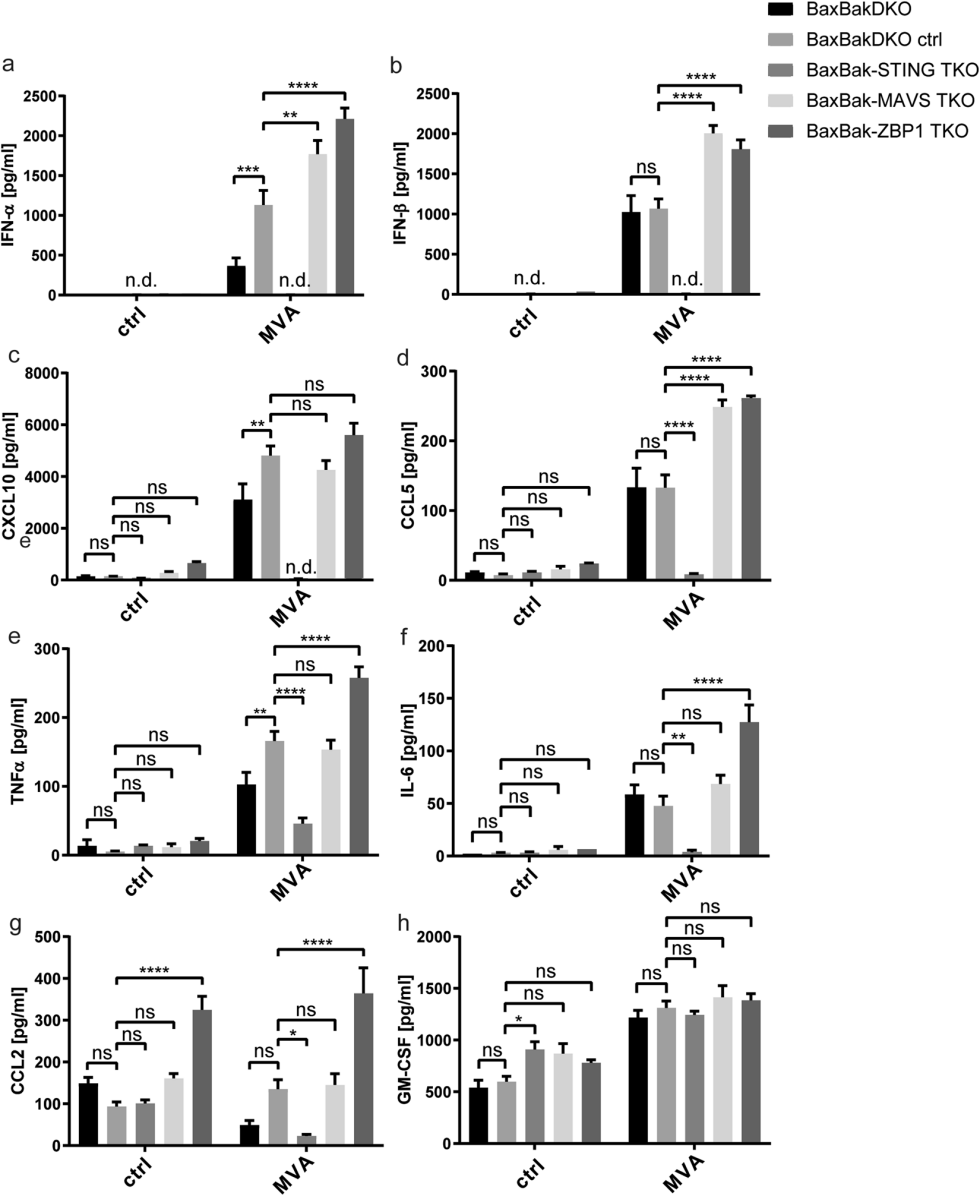
Influence of DNA recognition pathways on macrophage activation. **a**–**h** Bax/Bak-STING TKO, Bax/Bak-MAVS TKO, or Bax/Bak-Zbp1 TKO cells (genetically modified by CRISPR/Cas) were infected with MVA at an MOI of 2 and supernatants were collected after 22 h. Samples were analysed for the cytokines indicated by a bead-based assay (Legendplex, antivirus response panel, Biolegend). Bax/BakDKO and Bax/Bak ctrl cells (expressing an irrelevant gRNA directed against EGFP) served as controls. Shown are the means/SEM of *n* = 3 independent experiments. *p < 0.05; **p < 0.01, ***p < 0.001, ****p < 0.0001; ns nonsignificant (*p* ≥ 0.05).

We further addressed the contribution of individual DNA recognition pathways on inflammatory cytokine responses. STING-deficiency (but not DAI/Zbp1- or MAVS-deficiency) severely compromised secretion of all cytokines analyzed except GM-CSF (Fig. 7a–h). Most strikingly, STING-deficiency abrogated MVA-induced IFN-I-secretion and secretion of CXCL10 and CCL5 (Fig. 7a–d), and strongly reduced IL-6-secretion (Fig. 7f). CCL2-secretion was partially STING-dependent (Fig. 7g). In contrast, both MAVS and DAI/Zbp1-deficiency increased the IFN-I response upon MVA-infection (Fig. 7a, b, d). DAI/Zbp1-deficiency also led to increased TNF and IL-6-secretion. Viral protein expression seemed to be slightly increased in Bax/Bak- or STING-deficient cells as well as in the presence of QVD and Nec-1 (Fig. S11). The data suggest that MVA is recognized by a number of PRRs in macrophages, which both trigger the induction of cytokines and activate apoptosis-pathways.

All analyses so far were done with the attenuated VACV-derivative MVA. The non-attenuated VACV has a number of additional genes which are lost in the MVA strain. Various viral genes are known to interfere with the cell death machinery, and some, like CrmA, may not be functional in MVA [27]. In order to test for possible differences in cell death-induction compared to non-attenuated VACV, we included a VACV-strain (Western Reserve).

Total cell death rates of macrophages infected with VACV were comparable to infection with MVA (Fig. S12a). Apoptotic cell death strongly depended on mitochondrial apoptosis, and Nec-1 alone efficiently blocked overall cell death in the absence of Bax/Bak (Fig. S12a, b). In contrast to MVA-infection, mitochondria-independent apoptosis played no substantial role. The results indicate that in VACV-infection, a deficiency in mitochondrial apoptosis shifts cell death to RIPK1-dependent necroptosis instead of caspase-8-dependent apoptosis.

## Discussion

**Fig. 8:**
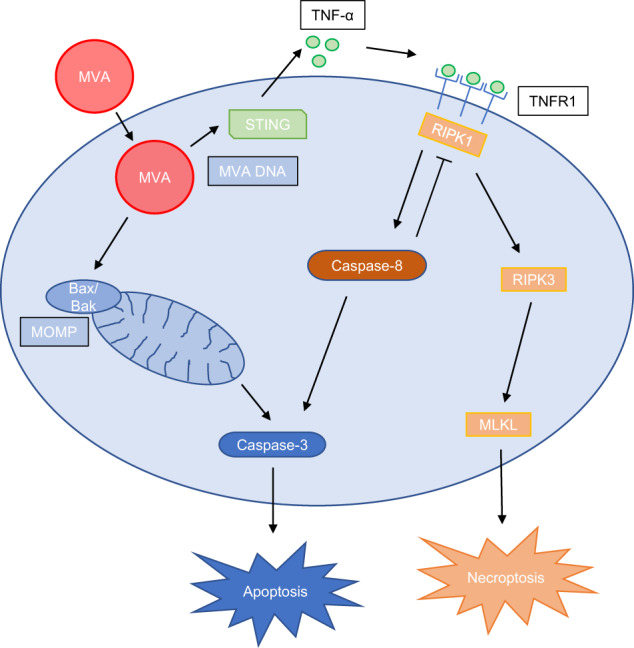
Schematic overview of cell death-signaling induced by MVA-infection in murine macrophages. MVA infection in macrophages activates various signaling pathways which include both innate immune responses and cell death signaling. Recognition of MVA in the cell leads to STING-dependent TNF secretion. Cell death signaling pathways activated by MVA comprise Bax/Bak-dependent mitochondrial apoptosis and extrinsic apoptosis via STING-mediated autocrine/paracrine TNF signaling. In the absence of caspase activity, cell death shifts to RIPK3/MLKL-dependent necroptosis. Inhibition of individual pathways leads to compensatory death through the other pathways. (MOMP, mitochondrial outer membrane permeabilization).

In macrophages, MVA-induced mitochondrial apoptosis and TNF-dependent apoptosis. When caspases were blocked, TNF induced necroptosis at the same level of cell death instead; pyroptosis did not appear to play a role. It is intriguing to observe that all three forms of cell death can fully compensate for each other: blocking only mitochondrial apoptosis (Bax/Bak-deficiency) did not reduce cell death, nor did isolated caspase-inhibition. Likewise, isolated inhibition of necroptosis by RIPK3-deletion or RIPK1-inhibition had no effect on total cell death. Thus, signals appear to be generated by a viral infection that can kill the cell through various different pathways (summarized in Fig. 8). How the choice is made, whether all pathways are triggered in all cells simultaneously, and if a hierarchy of cell death modalities exists, are unanswered questions. Similar flexibility and interconnection between apoptosis, necroptosis (and in that infection also pyroptosis) and their protective effect against intracellular infection has also recently been shown for infection of macrophages with the bacterial pathogen *Salmonella* [28, 29], and with viruses, e.g., influenza A [22, 29] or SARS-Cov-2 [30].

We observed RIPK1 phosphorylation at S166 [31–34] only in the presence of caspase-inhibition in MVA-infected macrophages. This is consistent with the role of caspase-8 as a switch between apoptosis and, when inhibited, necroptosis. MVA-induced caspase-dependent RIPK3-cleavage limits necroptosis and favors apoptosis in macrophages, but caspase-inhibition, in contrast to other cell types, does not prevent but shifts death to necroptosis. The importance of RIPK1/3 in the regulation of anti-poxviral immunity has been shown in mouse models in vivo [35, 36]. Kinase activities of RIPK1 and RIPK3 not only regulated virus-induced necroptosis but were required for control of VACV replication. RIPK1/3-mediated cell death-induction in macrophages thus overcomes virus-induced pro-survival signaling and thereby probably contributes to control of poxvirus infection.

Autocrine TNF-secretion could clearly contribute to cell death, but the observed amounts cannot explain the strong TNF-dependent cytotoxic effects in MVA-infected cells. Importantly, treatment with Nec-1 or loss of RIPK3 did not block TNF-production, as has been seen in certain stimuli in macrophages [37], showing that the death-inducing effect of RIPK1 and RIPK3 is rather at the level of caspase-8 and MLKL-activation. The observed depletion of cIAP1/TRAF2 can however explain the striking cell death sensitivity to TNF because lack of these proteins permits strong caspase-8-activation through TNFR1 [38]. A possible reason for cIAP1/TRAF2-depletion in macrophages could be signaling through TNFR2 [39]. Oligomerized TNFR2 recruits TRAF2 along with TRAF1 and cIAP1/2, leading to NF-κB activation, but also depletes TRAF2-cIAP1/2 complexes in the cytoplasm [40, 41]. The largely TNF-independent cIAP1/TRAF2-depletion in our system however pointed to a TNFR2-independent mechanism. Other TNF superfamily members may be involved here. TWEAK, which is widely expressed in monocytes/macrophages and is associated with inflammation [42], can promote lysosomal degradation of cIAP1/TRAF2 in tumor cells [43]. TWEAK/Fn14 might thus contribute to sensitization to TNF-mediated cell death. Moreover, the observed small contribution of Bax/Bak to cIAP1/TRAF2-depletion may point to a minor role of factors such as Smac/DIABLO or Omi/HtrA2 released from mitochondria in the modulation of caspase-8-activation.

Upstream mechanisms of Bax/Bak activation in MVA-infected macrophages may be explained by a virus-induced shutdown of host genes, resulting in loss of short-lived antiapoptotic Bcl-2-family members, particularly Mcl-1. This may be additionally triggered by upregulation of proapoptotic BH3-only proteins, especially Noxa, as has previously been observed during MVA-infection in other cell types [8, 13].

The mode of cell death of infected epithelial cells, but similarly of innate immune cells, may affect subsequent events of the innate immune response. In MVA-infected macrophages, we observed a clear silencing of IFN-I-responses by caspases. It has been reported before that caspases can reduce the induction of IFN-I, which otherwise is the result of mitochondrial membrane permeabilization [44, 45]. IFN-I-production during influenza A-infection of human monocytes was also enhanced by caspase-inhibition [46]. It has been suggested that mitochondrial DNA (mtDNA) is released during apoptosis and triggers cytokine secretion through recognition by cGAS/STING [44, 45, 47]. Loss of STING abrogated the secretion of IFN-I and IFN-dependent factors and reduced TNF-secretion in our study. Although this would be consistent with the mtDNA-dependent cGAS-stimulation, the reported activation of cGAS during apoptosis also depended on Bax/Bak [44, 45], but Bax/Bak were not required for STING-mediated cytokine-secretion in MVA-infection. The results suggest that STING-dependent cytokine-secretion was primarily independent of apoptosis and may occur upon recognition of viral DNA in the cytosol.

RIPK3-deficiency interfered with the secretion of certain cytokines upon MVA-infection as well as with constitutive CCL2-secretion. This is intriguing because this effect was not linked to different levels of cell death. RIPK3-deficiency had an impact on the production of cytokines and led to reduced neuroinflammation upon West Nile virus infection, independent of cell death-signaling [48]. It is possible that RIPK3-signals are required for cytokine-expression as has been previously shown [37, 49], but because RIPK3 can also regulate MLKL-dependent vesicular secretion [49, 50], this may further contribute to cytokine-secretion.

Our results support a complex view of cell death as a response to viral infection but also as a regulator of immune cell activation. The immunogenicity of a virus likely depends not only on its PAMPs but also on its ability to induce or inhibit individual cell death modalities. TNF is generally considered a weak inducer of cell death outside experimental modulation of signaling. In viral infection, cell death-induction through TNF seems to be an important facet of its pleiotropic signaling qualities.

## Material and methods

### Cell lines, cell culture, and viral infection

HoxB8 macrophage progenitors were derived from the bone marrow of C56BL/6 wild type (wt) or genetically modified Bax^−/−^Bak^−/−^, TNF^−/−^, Ripk3^−/−^, Caspase-1^−/−^, and Caspase-1^−/−^11^−/−^ mice. Polyclonal macrophage progenitor cell lines were established by retroviral transduction of Hoxb8 and selection in the presence of GM-CSF as described by Wang et al. [16]. Immortalized progenitor cells were cultured in non-cell culture treated six well-plates in VLE RPMI 1640 (with stable Glutamine, with 2.0 g/L NaHCO_3_, Biochrom, or PAN) supplemented with 10% FCS (Gibco, Thermo Fisher) in the presence of 5 µM estrogen and 1% GM-CSF supernatant from GM-CSF-producing B16 cells corresponding to a final GM-SCF concentration of ~10 ng/ml according to Wang et al. [16] (kindly provided by Hans Häcker). Cells were kept at a maximum density of 0.5–1 × 10^6^ per well with 3 ml of medium. Cells were split every 2–3 days or the day before seeding them for an experiment or starting differentiation. Cell viability and density were assessed with the CASY cell counter (Omni Life Science). Cells were kept in culture for a maximum of 4 to 6 weeks before using a new frozen batch of the same cell line. Differentiation was induced by estrogen removal and culture in a medium containing 1% GM-SCF supernatant for 7 days (0.5 × 10^6^ progenitor cells in 10 cm dishes in 10 ml). An additional amount of 50 µl of GM-CSF supernatant was added to the plate on day 3 or 4 if necessary. Differentiated cells were washed once in PBS and harvested by accutase treatment for 15 min at 37 °C.

Human primary monocytes were isolated from peripheral blood from a healthy voluntary donor by negative selection using the EasySep^TM^ direct human monocyte isolation kit (StemCell Technologies) according to the manufacturer’s instructions. The purity of isolated cells (>95% CD14^+^) was confirmed by flow cytometry staining of surface markers using anti-human CD14-PE (clone M5E2, StemCell Technologies) and anti-human CD45-APC (clone HI30, StemCell Technologies) (Fig. S4e). For experiments, cells were cultured in VLE RPMI 1640 (with stable Glutamine, with 2.0 g/L NaHCO3, PAN) supplemented with 10% heat-inactivated FCS (Gibco, Thermo Fisher), 10 ng/ml GM-CSF (Peprotech), 1% Pen/Strep, and 1 mM sodium pyruvate (Gibco, Thermo Fisher).

HEK293FT cells (Invitrogen, Carlsbad, CA, USA) were cultured in tissue-culture treated plates in DMEM supplemented with 10% FCS and were used for lentivirus production.

HeLa229 cells (ATCC Cat# CCL2.1) were cultured in tissue-culture treated plates in RPMI (Gibco, Thermo Fisher) supplemented with 10% FCS.

The inhibitors QVD-OPh (QVD) (Apex Bio, Gentaur), ZVAD-fmk (ZVAD) (Gentaur), necrostatin 1 (Nec-1) (Sigma-Aldrich), ABT-737 (Selleckchem), S63845 (Selleckchem), and TL32711 (Birinapant, Active Biochem) were used as indicated.

### Monitoring of cell differentiation by Giemsa staining and cell surface marker expression

Staining for cellular and nuclear morphology was performed on cytospins from cultures of progenitors or differentiated macrophages by incubating with Giemsa solution (Merck, Darmstadt, Germany) after methanol fixation. Analysis by brightfield microscopy was performed using a Keyence BZ9000 microscope at a magnification of 20x (Keyence, Neu-Isenburg, Germany).

Expression of cell surface markers was measured after blocking of unspecific binding sites with CD16/CD32 Fc-Block (BD Biosciences, San Jose, CA, USA). Cells were stained with anti-CD11b-PE (clone M1/70, #12-0112, eBioscience, San Diego, CA, USA), anti-CD11c-PE (clone HL3, #553802, BD), or anti-F4/80-AF647 (clone A3-1, #MCA497A647, Biolegend, San Diego, CA, USA) followed by flow cytometry analysis on a FACS Calibur (BD Biosciences, Heidelberg, Germany).

### Generation of primary mouse bone marrow-derived macrophages (BMDMs)

BMDMs were generated by incubating mouse bone marrow cells in BMDM medium (RPMI 10% FCS, 1% penicillin/streptomycin supplemented with 50 ng/ml M-CSF (Peprotech) for 7 days. Mice were sacrificed by neck dislocation and bone marrow cells were isolated by flushing the tibias and femurs. After removal of erythrocytes using red blood cell lysing buffer (Sigma), bone marrow cells were frozen in liquid nitrogen and thawed immediately prior to use. Cells were seeded in a 10 cm petri dish at 15–30 × 10^6^ per plate in RMPI supplemented with 10% FCS, 1% penicillin/streptomycin, and 50 ng/ml M-CSF. On day 4, 10 ml of fresh media was added. Cells were harvested on day 7 by washing once with PBS and then incubating with accutase for 20 min at 37 °C, collecting only the adherent cells. Infection with MVA was performed as described above with HoxB8 macrophages.

### Virus infections

For MVA (kindly provided by Gerd Sutter, Munich), or VACV (strain Western Reserve, kindly provided by Peter Aichele, Freiburg) infections, 0.5 × 10^6^ differentiated macrophages were seeded in a 12-well plate in 500 µl complete medium. MVA or VACV was diluted in complete medium, vortexed, and sonicated (three cycles, 30′ each, Bioruptor System, Diagenode). Infection was conducted in a total volume of 1 ml by adding an equal volume (500 µl) of virus suspension to the cells.

### CRISPR/Cas9-mediated genome editing

For CRISPR/Cas9-mediated genome editing, the following sgRNA primer sequences were used: RIPK3, CACCGGGAACCGCTGACGCACCAGT; MLKL, CACCGGCACACGGTTTCCTAGACGC; DAI/Zbp1, CACCGCAGGTGTTGAGCGATGACGG;

STING, CACCGCACCTAGCCTCGCACGAACT; MAVS, CACCGAATAATCTCCAGCGCCGGCC.

sgRNA sequences were cloned into the lentiCRISPRv2-puro vector (Addgene) (expressing both the Cas9 protein and the sgRNA) according to Sanjana et al. [51]. CRISPR/Cas9 constructs were stably introduced into macrophage progenitors by lentiviral gene transfer. For lentivirus production, expression vectors were transfected into HEK293FT cells, together with packaging plasmids psPAX2 and pMD2.G using Fugene HD (Roche, Mannheim, Germany). psPAX2 and pMD2.G were gifts from Didier Trono (Addgene plasmids #12260 and #12259). Lentiviral supernatants were harvested on days 2 or 3, filtered, and transduced at a cell density of 0.5–1 × 10^5^/ml in the presence of 5 μg/ml polybrene. Transduced cells were subjected to puromycin selection (7.5 µg/ml) to select polyclonal knockout cell lines. Knockout efficiency was confirmed by Western blotting (Fig. S8).

### Apoptosis and cell death assays

For staining of active caspase-3, cells were washed with PBS, fixed in 2% paraformaldehyde, and permeabilized with 0.5% saponin (Sigma-Aldrich). Cells were incubated with anti-active caspase-3 (BD Pharmingen, Heidelberg, Germany) in PBS/0.5% BSA/0.5% saponin for 20 min, stained with anti-rabbit-Alexa-Fluor647 (Dianova GmbH, Hamburg, Germany) for 20 min, and analyzed by flow cytometry.

AnnexinV-propidium iodide staining was done by washing cells with annexin V-binding buffer (eBioscience) and staining with AnnexinV-FITC (1: 20; BD Pharmingen) 15 min at 4 °C. Propidium iodide (1 μg/ml; Sigma-Aldrich) was added and cells were analyzed on a FACS Calibur (Becton Dickinson, Heidelberg, Germany). The rate of dead cells was determined as a percentage of Annexin5/PI-positive cells (cells positive for annexin V or PI or both) of all cells analysed.

In some experiments, a fixable live-dead stain (LIVE/DEAD Fixable Far-Red Dead Cell Stain, Life Technologies, Carlsbad, USA) according to the manufacturer’s protocol was combined with staining for active caspase-3. After live-dead staining, samples were fixed in 4% paraformaldehyde for 30 min at RT, washed with PBS, and further subjected to the caspase-3 staining protocol as described above. Samples were analysed by flow cytometry on a FACS Calibur.

### Immunoblot analysis

Cells were harvested with accutase treatment (20 min 37 °C), washed with PBS, lysed directly in Laemmli buffer, and boiled at 95 °C for 5 min. Lysis was done at a concentration of 0.625 × 10^6^ cells/100 µl Laemmli buffer for differentiated cells and of 5 × 10^6^ cells/100 µl Laemmli buffer for progenitors. Extracts were separated by SDS-PAGE on 4–20% Novex Tris-glycine gels (Life Technologies) or using the TGX™ FastCast™ Acrylamide Kit (12%, Biorad). Proteins were transferred onto PVDF membranes (0.2 μm) by wet transfer overnight at 250 mA or by Turboblot transfer (Biorad). Membranes were probed with antibodies against RIPK1 (BD Transduction Laboratories, clone 38/RIP, #610459), phospho-RIPK1 (S166, Cell Signaling, #31122), RIPK3 (ProSci, #2283), mouse caspase-8 (Cell Signaling, #4927 and #9429), human caspase-8 (#9746, Cell Signaling), mouse caspase-9 (#9504, Cell signaling), human caspase-9 (#9502, Cell Signaling), cleaved caspase-3 #9661, Cell Signaling), caspase-3 (#9662, Cell Signaling), phospho-MLKL (Ser345, clone D6E3G, Cell Signaling), MLKL (clone 3H1, Merck/Millipore), cIAP1 (clone 1E1-1-10, Enzo), TRAF2 (#4724, Cell Signaling), Caspase-1 (clone Casper-1, Adipogen, #AG-20B-0042), DAI/Zbp1 (Adipogen, clone Zippy-1, #AG-20B-0010), MAVS (Cell Signaling, #4983), STING (Cell Signaling, clone D2P2F, #13647), and GAPDH (#MAB384, Merck/Millipore). Proteins were visualized using peroxidase-conjugated anti-rabbit (Sigma), anti-mouse (Dianova), anti-hamster (Dianova), or anti-rat (NEB Cell Signaling) antibodies by enhanced chemiluminescence detection (ECL Prime, GE Healthcare, Dornstadt, Germany; SuperSignal West Femto or Pico Substrate, Pierce, Fisher Scientific, Schwerte, Germany). In some cases, blots were stripped before reprobing using the Restore™ PLUS Western Blot Stripping Buffer (Thermo Fisher Scientific).

### Quantification of cytokine-secretion in supernatants

Cytokine-secretion of macrophages was analysed in supernatants using a bead-based assay (LEGENDplex™ Mouse AntiVirus Response Panel (13-plex), Biolegend) according to the manufacturer’s instructions. Samples were analysed by flow cytometry on a FACS Fortessa (Becton Dickinson). In some experiments, secretion of TNF by macrophages was quantified by Enzyme-linked immunoabsorbent assay (ELISA) using the Mouse TNF alpha ELISA Ready-SET-GO!^®^ kit (eBioscience, Thermo Scientific) according to the manufacturer’s instructions. Absorption was analysed on a multi-plate-reader (Tecan).

### Statistical analysis

Statistical analysis was performed with Prism (V8, GraphPad) using two-way ANOVA and Sidak’s or Tukey’s multiple comparison test for multiple testing. Bars represent the mean and error bars show the standard error of the mean. At least three independent biological replicates were chosen as the sample size for each analysis.

## Supplementary information

Supplementary figures S1-S12

## Data Availability

The datasets used and/or analyzed during the current study are available from the corresponding author on reasonable request.
