# Supplementary material for: Diversity of cell death signaling pathways in macrophages upon infection with modified vaccinia virus Ankara (MVA)
Source: Cell Death Dis. 2021 Oct 28;12(11):1011. doi: 10.1038/s41419-021-04286-3 (PMC8551665; doi:10.1038/s41419-021-04286-3)
Supplement: Supplementary file 1 — Supplementary figures S1-S12 [file 41419_2021_4286_MOESM1_ESM.pdf]

Supplementary figure legends

Figure S1 *Typical* m*orphology and surface marker expression of progenitors and differentiated Hoxb8 macrophages*

a, Hoxb8 progenitors and d7 differentiated macrophages of WT, Bax/Bak DKO and Bax/Bak DKO ctrl lines were subjected to cytospin onto glass slides, fixed with methanol and stained with Giemsa. Images were taken by bright field microscopy with a BZ900 microscope (Keyence).

b, Hoxb8 progenitors and d7 differentiated macrophages of WT, Bax/Bak DKO and Bax/Bak DKO ctrl lines were stained for various myeloid surface markers (CD11b, CD11c, F4/80) and analysed by flow cytometry (FACS Calibur).

Fig. S2 *Cell death analysis in MVA-infected macrophages by flow cytometry*

a,b, D7 differentiated wt or Bax/Bak-DKO Hoxb8 macrophages were infected with MVA at a MOI of 2 and harvested after 22h by accutase treatment followed by annexin/PI staining (a) or active caspase-3 staining (b) and flow cytometry analysis (FACS Calibur). Shown are single dot plots representative of 6 (a, annexinV/PI) or 5 (b, active caspase-3) experiments.

Fig. S3 *Viability analysis of single inhibitor controls*

D7 differentiated Hoxb8 macrophages of the genotypes indicated were incubated with the single or combined inhibitors QVD (20µM), Nec-1 (10µM) or ZVAD (20µM) for 22 h. Cells were harvested by accutase treatment, stained for annexinV/PI (a) or active caspase-3 (b) and analysed by flow cytometry (FACS Calibur). Shown are means/SEM of 2-6 independent experiments. Statistical analysis has been performed for wt samples. ****p<0.0001; ns, non-significant (p>=0,05).

Fig. S4 *Analysis of MVA-induced cell death in primary human monocytes*

a-d, Human peripheral blood-derived monocytes were infected with MVA at a MOI of 2 as indicated (a,b) or left uninfected (c,d). Cells were harvested at 22 h by accutase treatment. Inhibitors Nec-1 (10µM) and/or QVD (20µM) were used where indicated. Cells were stained for annexinV/PI (a,c) or active caspase-3 (b,d) and analysed by flow cytometry (FACS Calibur). Shown are means/SEM of 3 independent experiments. ***p<0,001; ****p<0.0001; ns, non-significant (p>=0,05).

e, Confirmation of purity of human monocytes by surface marker staining. Directly after isolation, cells were stained with anti-CD14-PE and anti-CD45-APC (right panel) or left

unstained (left panel) and analysed by flow cytometry (FACS Calibur). Samples are pregated on live cells. Data are representative for 3 independent experiments.

Fig. S5 *Western blot analysis of cell death-related proteins in WT and BaxBakDKO macrophages upon MVA infection*

a-e, D7 differentiated WT or BaxBak-DKO Hoxb8 macrophages (a,b,c) or primary murine bone marrow-derived macrophages (d,e) were infected with MVA at a MOI of 2 and harvested at the indicated time points by accutase treatment. Cells were lysed in Laemmli sample buffer and samples were boiled at 95°C for 5 min. Samples were subjected to SDS-PAGE, transferred onto PVDF membranes and probed with the antibodies indicated. The inhibitors Nec-1 (10µM) and QVD (20µM) were used as indicated. Treatment with a combination of TNF (10ng/ml)/Birinapant (10µM)/Z-VAD (20µM, added 30 min prior to other stimulants) for 6 hours was included as positive control for MLKL phosphorylation. GAPDH served as loading control. * Asterisks indicate unspecific bands. Blots are representative for 3 (a,b,c) or 2 (d,e) independent experiments each.

Fig. S6 *Analysis of caspase cleavage profile and cell death in HeLa human epithelial cells*

a, HeLa cells were infected with MVA at a MOI of 2 and harvested 18 hours post infection by direct lysis in Laemmli buffer. Samples were boiled at 95°C for 5 min and subjected to SDS-PAGE, transferred onto PVDF membranes and probed with the antibodies indicated. Combined treatment with ABT-737 (1µM) and S63845 (200µM) (A737/S63) was included as positive control. GAPDH served as loading control. Blots are representative for 3 independent experiments.

b,c, HeLa cells were infected with MVA at a MOI of 2 and harvested 18 hours post infection. Cells were stained for annexinV/PI (b) or active caspase-3 (c) and analysed by flow cytometry (FACS Calibur). Combined treatment with ABT-737 (1µM) and S63845 (200µM) (A737/S63) was included as positive control. Shown are means/SEM of 3 independent experiments.

Figure S7 *Influence of TNF stimulation on viability of non-infected macrophages*

D7 differentiated BaxBak-DKO ctrl Hoxb8 macrophages (expressing an irrelevant gRNA directed against EGFP) were stimulated with recombinant murine TNF (Peprotech) for 22h at the concentrations indicated. Cells were harvested by accutase treatment and cell death was determined by annexin/PI staining followed by flow cytometry analysis (FACS Calibur).

Figure S8 *Confirmation of CRISPR knockouts by Western blot*

a-e, Stable Hoxb8 progenitor lines deficient in the genes indicated were established on a Bax/Bak-deficient background by lentiviral CRISPR/Cas9-mediated knockout. Cells were directly lysed in Laemmli sample buffer, and whole cell lysates were boiled at 95°C for 5 min. Samples were subjected to SDS-PAGE on a 4-20% Novex Tris-glycine gel, transferred onto PVDF membranes and probed with the antibodies indicated. BaxBak ctrl cells (expressing an irrelevant control gRNA directed against EGFP) served as controls. GAPDH and β-actin served as loading controls. Where more than one gRNA was tested, knockouts expressing the following gRNAs were used for experiments: Zbp1: g1 (c); STING: g2 (d); MAVS: g1 (e).

Figure S9 *Impact of TNF or RIPK3 deficiency on the cytokine response of MVA-infected macrophages*

a-h, Supernatants of d7 differentiated WT (black bars), $TNF^{-/-}$ (light grey bars) or $Ripk3^{-/-}$(dark grey bars) Hoxb8 macrophages infected with MVA at a MOI of 2 were collected after 22h. Samples were analysed for the cytokines indicated by a bead-based assay (Legendplex, anti-virus response panel). Shown are means/SEM of 3 independent experiments. *p<0.05; **p<0.01; ns, non-significant (p>=0,05).

Figure S10 *Depletion of STING/TNF on a Bax/Bak-double-deficient background reduces caspase-8 cleavage and MLKL phosphorylation during MVA infection*

a,b, D7 differentiated Bax/Bak-DKO ctrl or Bax/Bak-STING-TKO Hoxb8 macrophages were infected with MVA at a MOI of 2 and harvested at the indicated time points by accutase treatment. For functional TNF depletion, Bax/Bak-DKO ctrl cells were additionally treated with a neutralizing anti-TNF antibody (1:1000) during infection. Cells were lysed in Laemmli sample buffer and samples were boiled at 95°C for 5 min. Samples were subjected to SDS-PAGE, transferred onto PVDF membranes and probed with the antibodies indicated. GAPDH served as loading control. Blots are representative for 3 independent experiments.

Figure S11 *Comparison of viral protein expression in wt and various gene-deficient macrophage*s

a, D7 differentiated WT or Bax/Bak/MLKL TKO Hoxb8 macrophages grown in ibidi µ-slides were infected with MVA at a MOI of 2 for 22 hours. Inhibitors Nec-1 (10µM) and QVD (20µM) were used where indicated. Cells were fixed with 2% formaldehyde and permeabilised with 0,5% saponine. Cells were stained for nuclear DNA (Hoechst) and with an anti-vaccinia virus antibody (ab35219, Abcam).

b, D7 differentiated WT or Bax/Bak/MLKL TKO Hoxb8 macrophages were infected with MVA at a MOI of 2 and harvested after 22 hours by accutase treatment. Inhibitors Nec-1 (10µM) and/or QVD (20µM) were used when indicated. Cells were lysed in Laemmli sample buffer, and whole cell lysates were boiled at 95°C for 5 min. Samples were subjected to SDS-PAGE on a 4-20% Novex Tris-glycine gel, transferred onto PVDF membranes and probed with an antibody against vaccinia virus (ab35219, Abcam). β-actin served as loading control.

Fig. S12 *Cell death induction in macrophages during vaccinia virus infection*

a,b, D7 differentiated WT (black bars) or Bax/Bak-DKO (grey bars) Hoxb8 macrophages were infected with vaccinia virus (VV), strain Western Reserve (WR) at a MOI of 2 and harvested after 22h by accutase treatment. Cell death was analysed by live-dead staining (Live Dead FarRed, Thermo Fisher) (a) or active caspase-3 staining (b) followed by flow cytometry analysis. Inhibitors were used at the following concentrations where indicated: QVD, 20µM; Nec-1, 10µM.

Shown are means/SEM of 3 independent experiments. *p<0.05; **p<0.01, ***p<0.001, ****p<0.0001; ns, non-significant (p>=0,05).

Figure S1

a

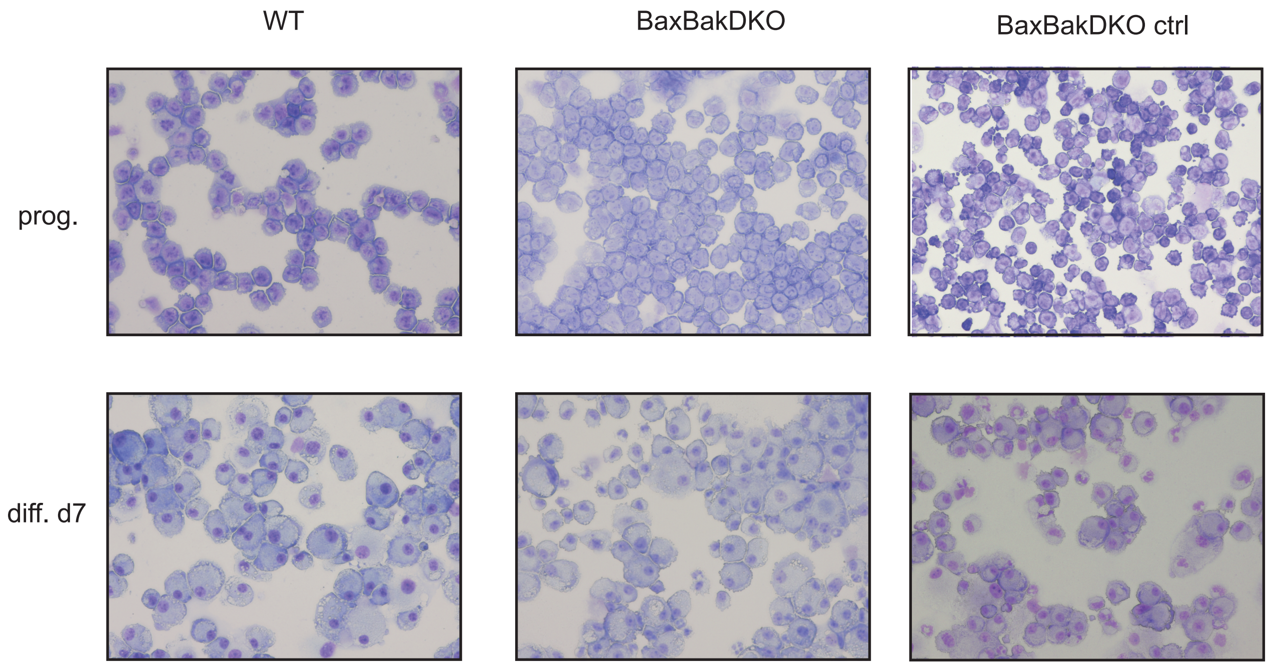
WT
BaxBakDKO
BaxBakDKO ctrl
prog.
diff. d7

b

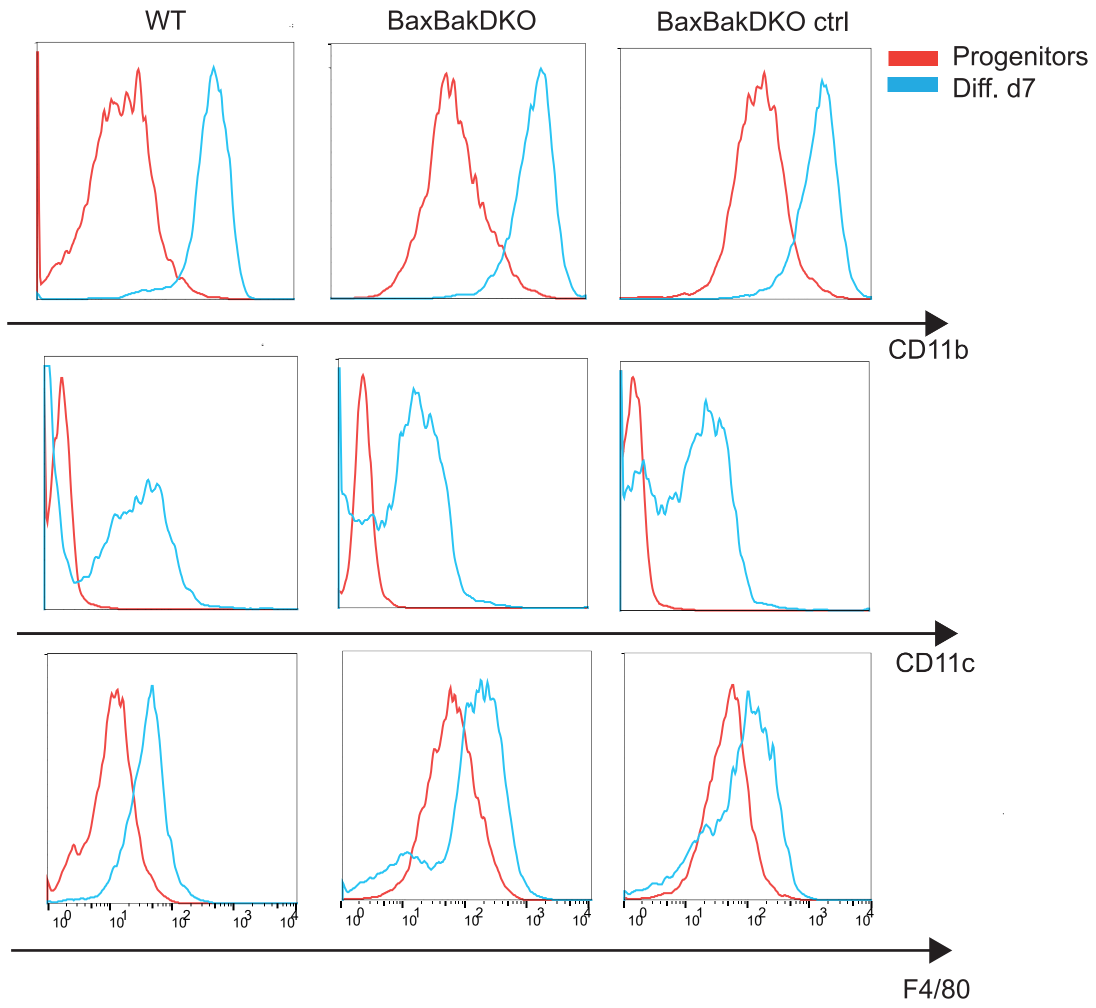
WT
BaxBakDKO
BaxBakDKO ctrl
Progenitors
Diff. d7
CD11b
CD11c
F4/80

Figure S2

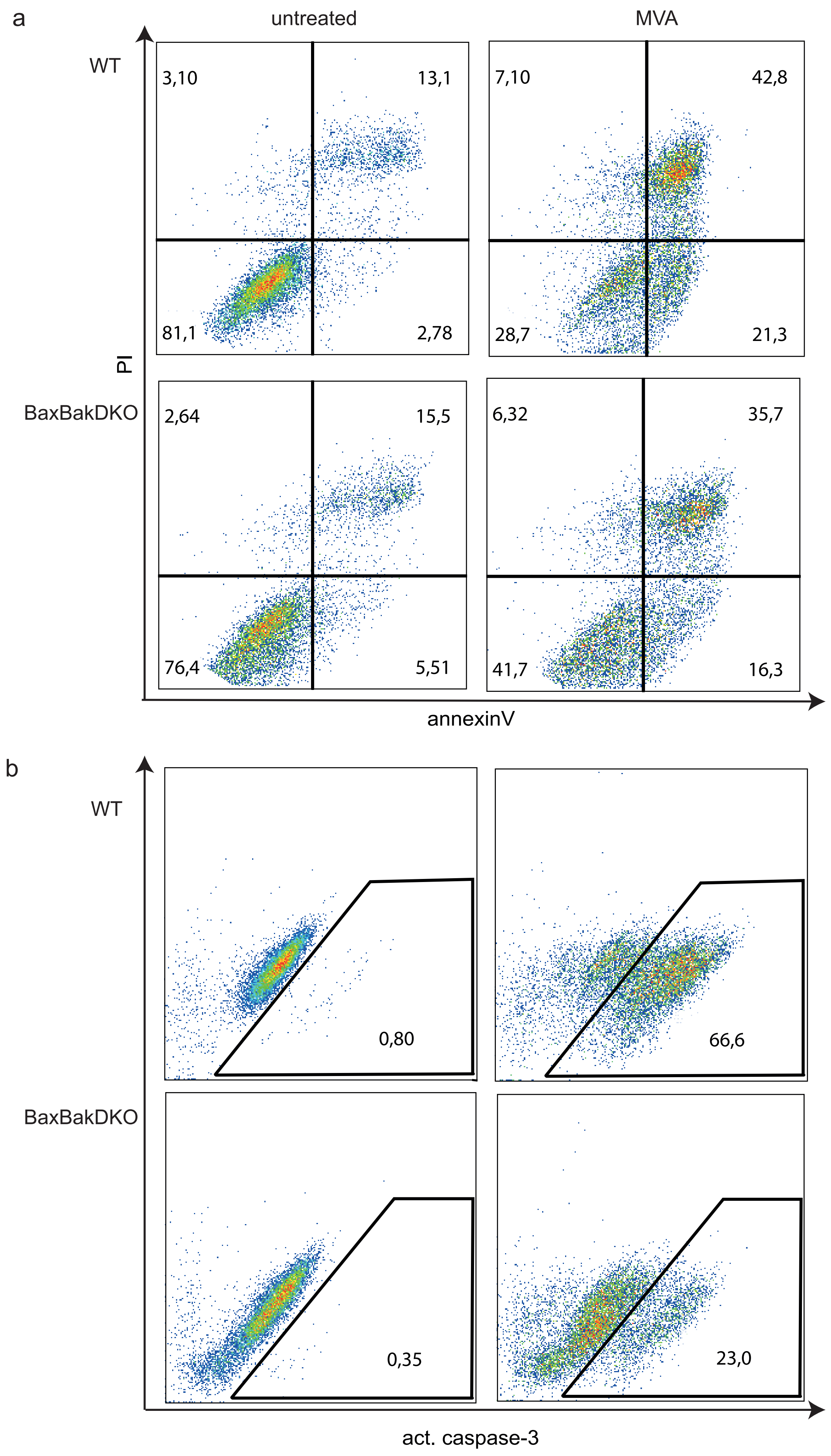
a
untreated
MVA
WT
PI
BaxBakDKO
3,10
13,1
81,1
2,78
7,10
42,8
28,7
21,3
2,64
15,5
76,4
5,51
6,32
35,7
41,7
16,3
annexinV
b
WT
BaxBakDKO
0,80
66,6
0,35
23,0
act. caspase-3

Figure S3

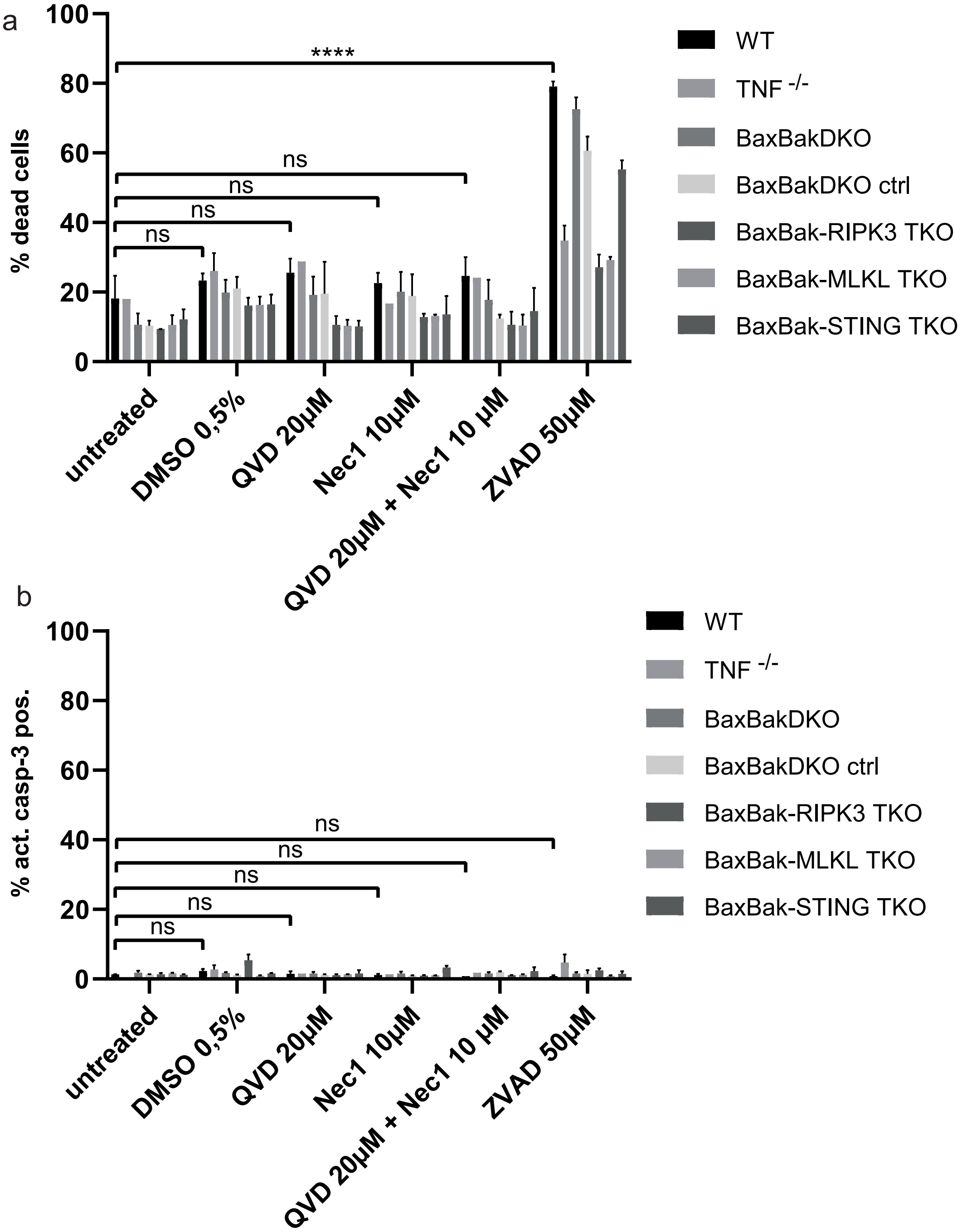
a
100
80
60
40
20
0
% dead cells
****
ns
ns
ns
ns
untreated
DMSO 0,5%
QVD 20µM
Nec1 10µM
QVD 20µM + Nec1 10 µM
ZVAD 50µM
WT
TNF -/-
BaxBakDKO
BaxBakDKO ctrl
BaxBak-RIPK3 TKO
BaxBak-MLKL TKO
BaxBak-STING TKO
b
100
80
60
40
20
0
% act. casp-3 pos.
ns
ns
ns
ns
ns
untreated
DMSO 0,5%
QVD 20µM
Nec1 10µM
QVD 20µM + Nec1 10 µM
ZVAD 50µM
WT
TNF -/-
BaxBakDKO
BaxBakDKO ctrl
BaxBak-RIPK3 TKO
BaxBak-MLKL TKO
BaxBak-STING TKO

Figure S4

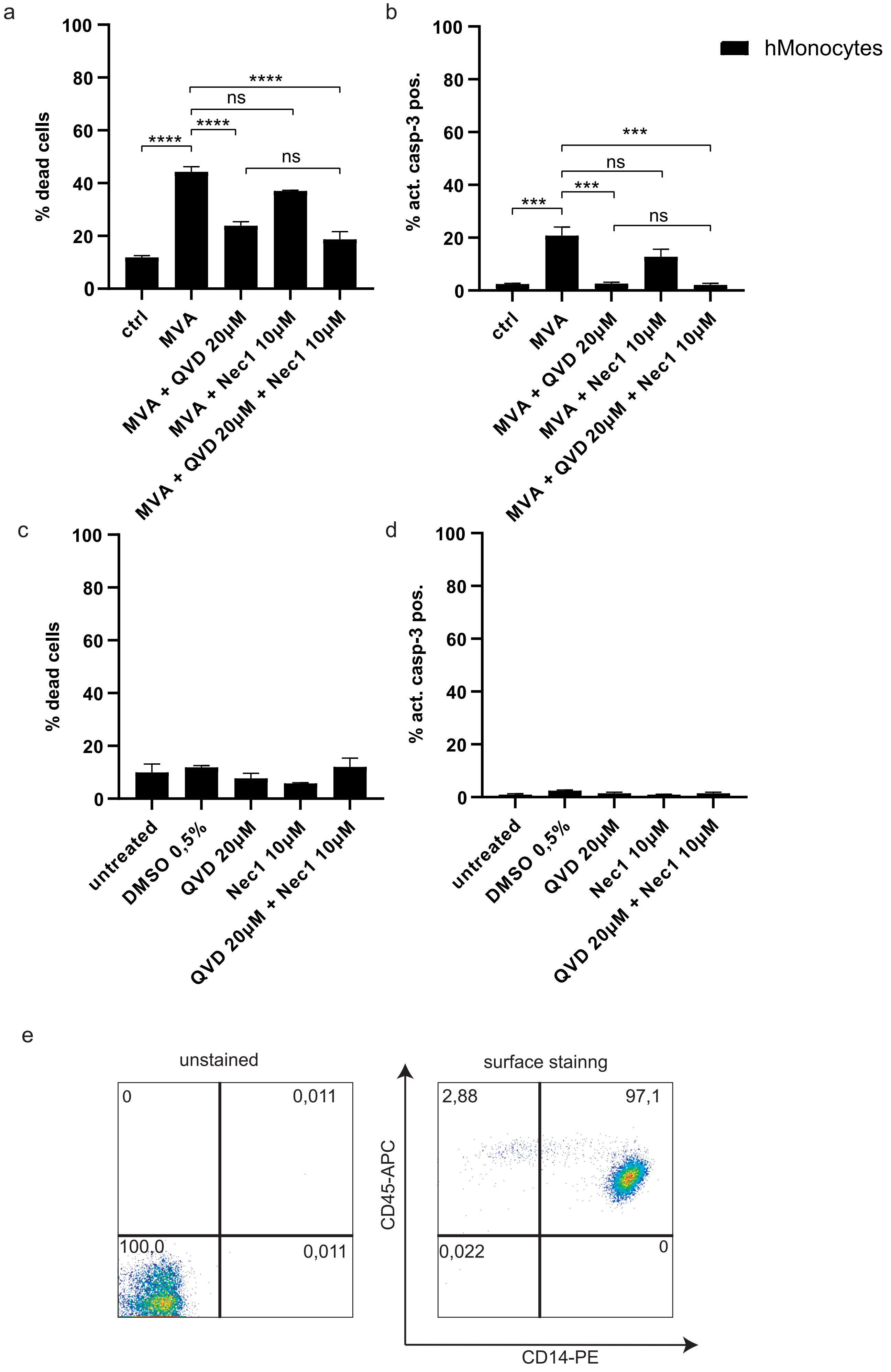
a
% dead cells
100
80
60
40
20
0
****
ns
****
****
ns
ctrl
MVA
MVA + QVD 20µM
MVA + Nec1 10µM
MVA + QVD 20µM + Nec1 10µM
b
% act. casp-3 pos.
100
80
60
40
20
0
hMonocytes
***
ns
***
***
ns
ctrl
MVA
MVA + QVD 20µM
MVA + Nec1 10µM
MVA + QVD 20µM + Nec1 10µM
c
% dead cells
100
80
60
40
20
0
untreated
DMSO 0,5%
QVD 20µM
Nec1 10µM
QVD 20µM + Nec1 10µM
d
% act. casp-3 pos.
100
80
60
40
20
0
untreated
DMSO 0,5%
QVD 20µM
Nec1 10µM
QVD 20µM + Nec1 10µM
e
unstained
0
0,011
100,0
0,011
surface stainng
2,88
97,1
0,022
0
CD45-APC
CD14-PE

Figure S5

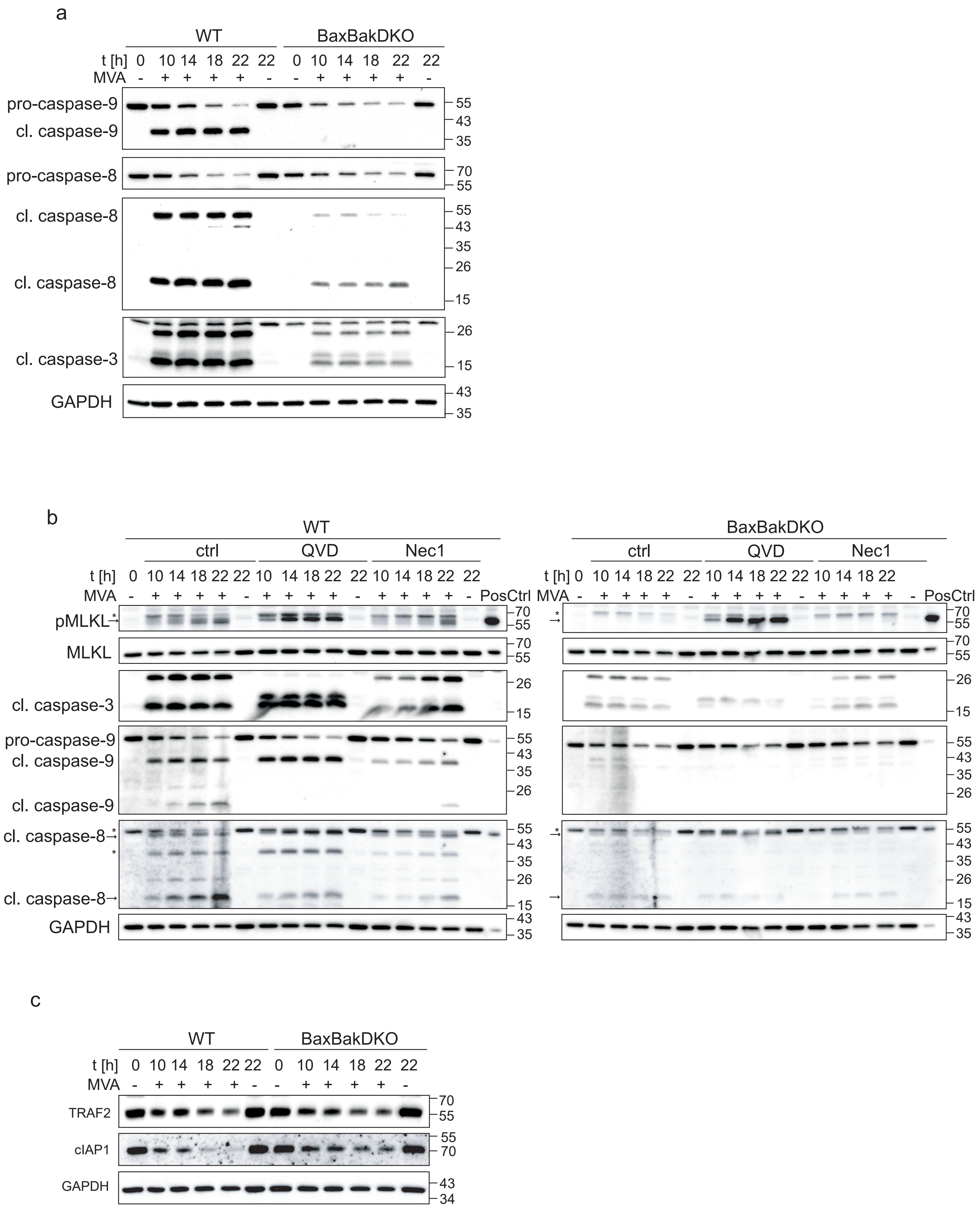

a
WT
BaxBakDKO
t [h] 0 10 14 18 22 22 0 10 14 18 22 22
MVA - + + + + - - + + + + -
pro-caspase-9
cl. caspase-9
pro-caspase-8
cl. caspase-8
cl. caspase-8
cl. caspase-3
GAPDH
b
WT
BaxBakDKO
ctrl
QVD
Nec1
t [h]
MVA
PosCtrl
pMLKL
MLKL
cl. caspase-3
pro-caspase-9
cl. caspase-9
cl. caspase-9
cl. caspase-8
cl. caspase-8
GAPDH
c
WT
BaxBakDKO
t [h] 0 10 14 18 22 22 0 10 14 18 22 22
MVA - + + + + - - + + + + -
TRAF2
cIAP1
GAPDH

Figure S5

d

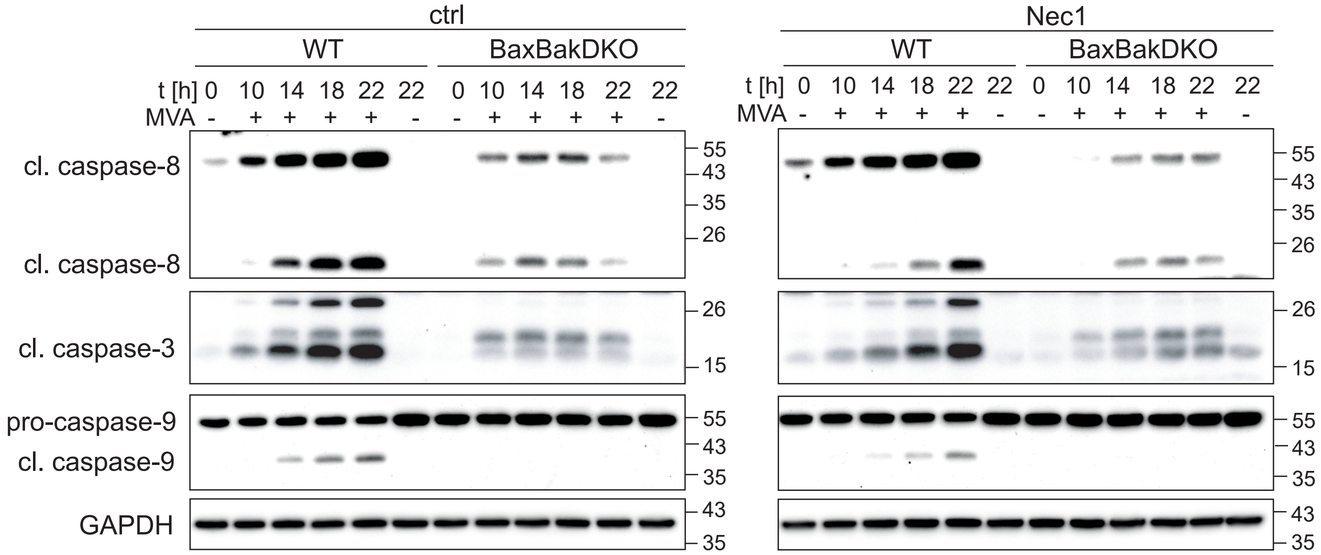

ctrl
Nec1
WT
BaxBakDKO
t [h]
MVA
cl. caspase-8
cl. caspase-8
cl. caspase-3
pro-caspase-9
cl. caspase-9
GAPDH
55
43
35
26
15

e

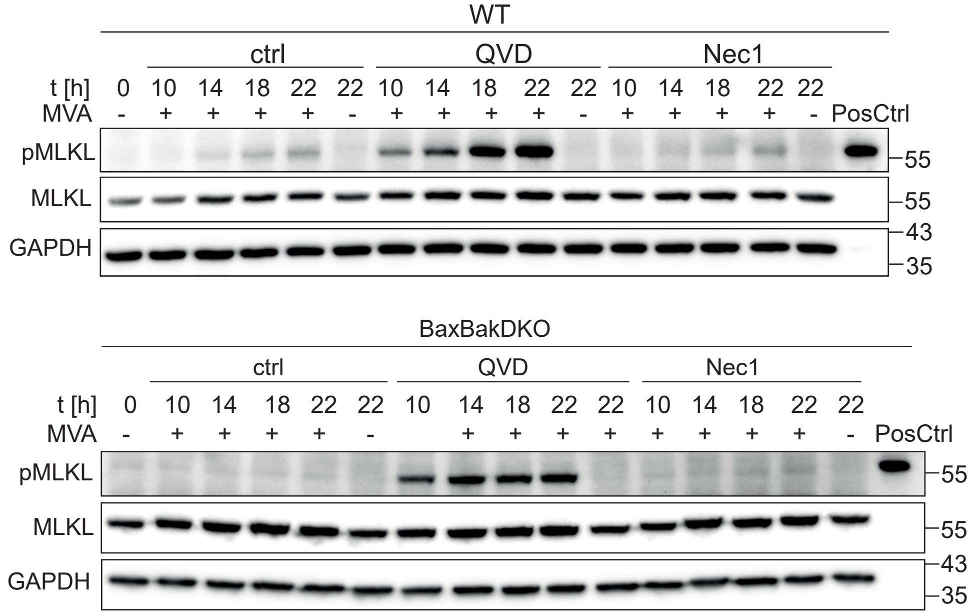

WT
BaxBakDKO
ctrl
QVD
Nec1
t [h]
MVA
PosCtrl
pMLKL
MLKL
GAPDH
55
43
35

Fig. S6

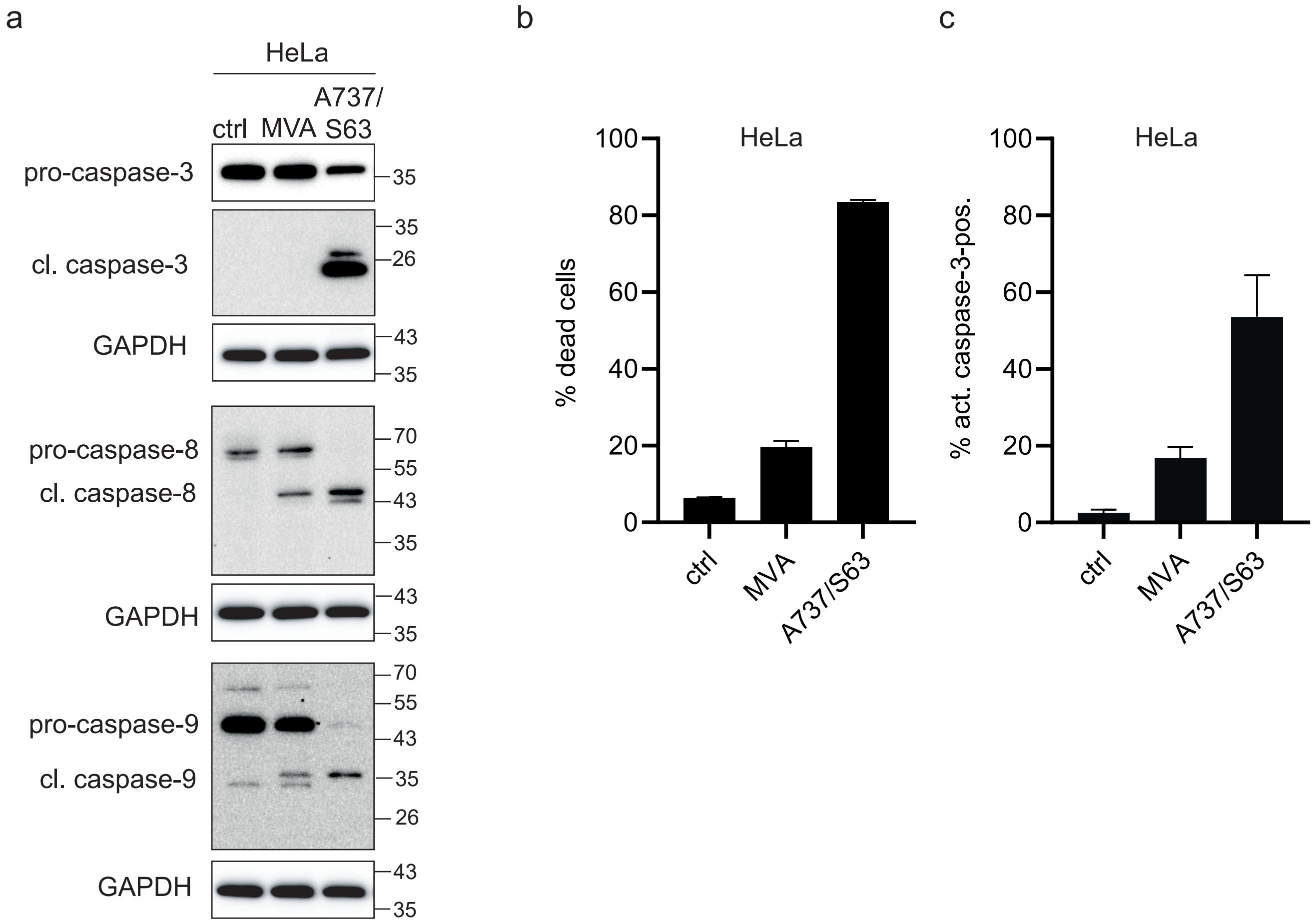

a
HeLa
ctrl
MVA
A737/
S63
pro-caspase-3
cl. caspase-3
GAPDH
pro-caspase-8
cl. caspase-8
GAPDH
pro-caspase-9
cl. caspase-9
GAPDH
35
26
43
70
55
b
HeLa
% dead cells
0
20
40
60
80
100
ctrl
MVA
A737/S63
c
HeLa
% act. caspase-3-pos.

Figure S7

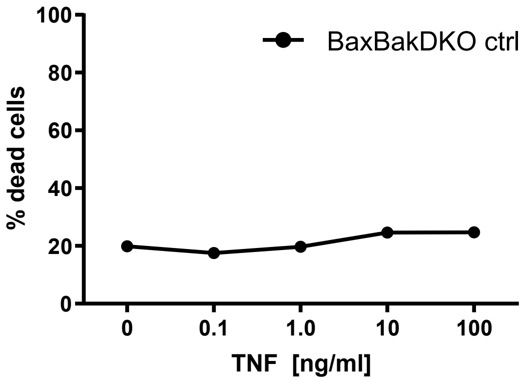

BaxBakDKO ctrl
% dead cells
100
80
60
40
20
0
0
0.1
1.0
10
100
TNF [ng/ml]

Figure S8

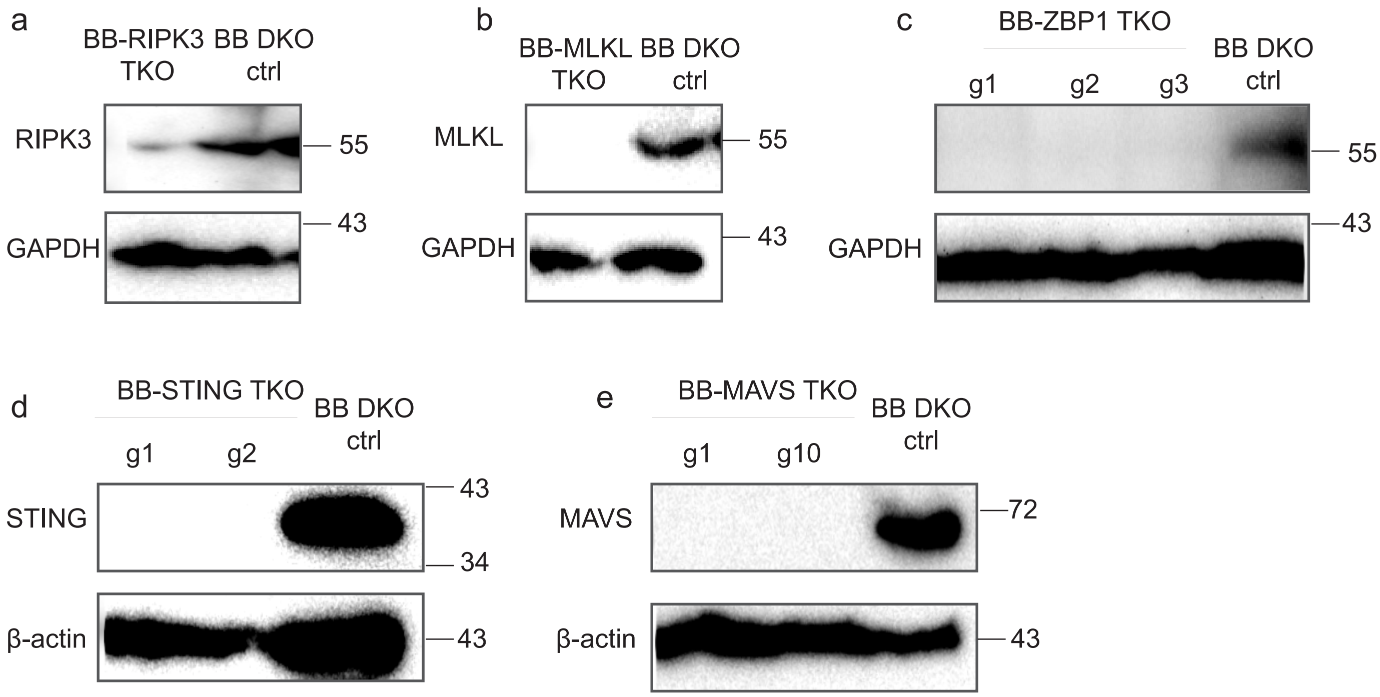
a
BB-RIPK3 TKO
BB DKO ctrl
RIPK3
55
GAPDH
43
b
BB-MLKL TKO
BB DKO ctrl
MLKL
55
GAPDH
43
c
BB-ZBP1 TKO
g1
g2
g3
BB DKO ctrl
55
GAPDH
43
d
BB-STING TKO
g1
g2
BB DKO ctrl
STING
43
34
β-actin
43
e
BB-MAVS TKO
g1
g10
BB DKO ctrl
MAVS
72
β-actin
43

Figure S9

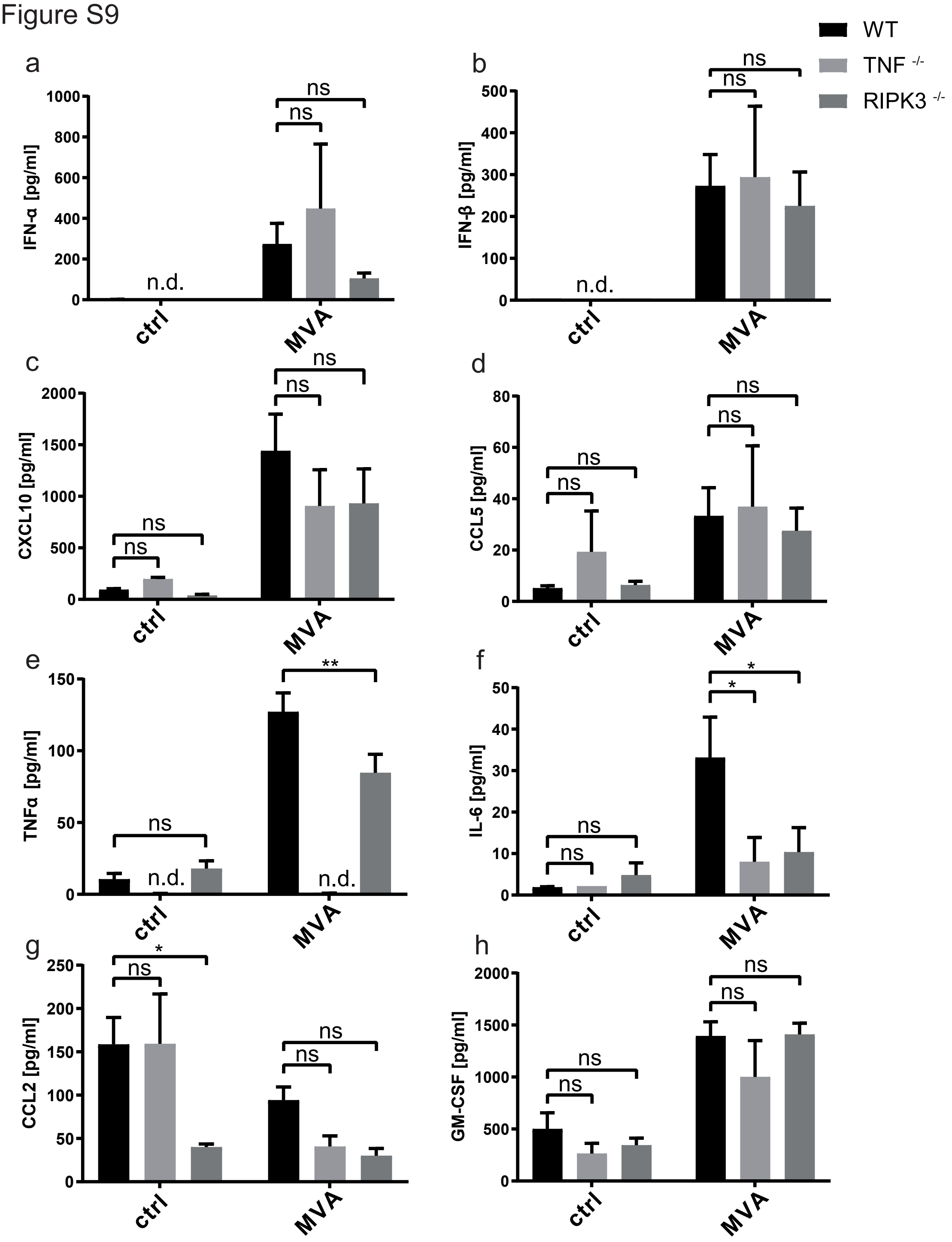

WT
TNF -/-
RIPK3 -/-
a
IFN-α [pg/ml]
b
IFN-β [pg/ml]
c
CXCL10 [pg/ml]
d
CCL5 [pg/ml]
e
TNFα [pg/ml]
f
IL-6 [pg/ml]
g
CCL2 [pg/ml]
h
GM-CSF [pg/ml]
ctrl
MVA
ns
n.d.
*
**

Figure S10

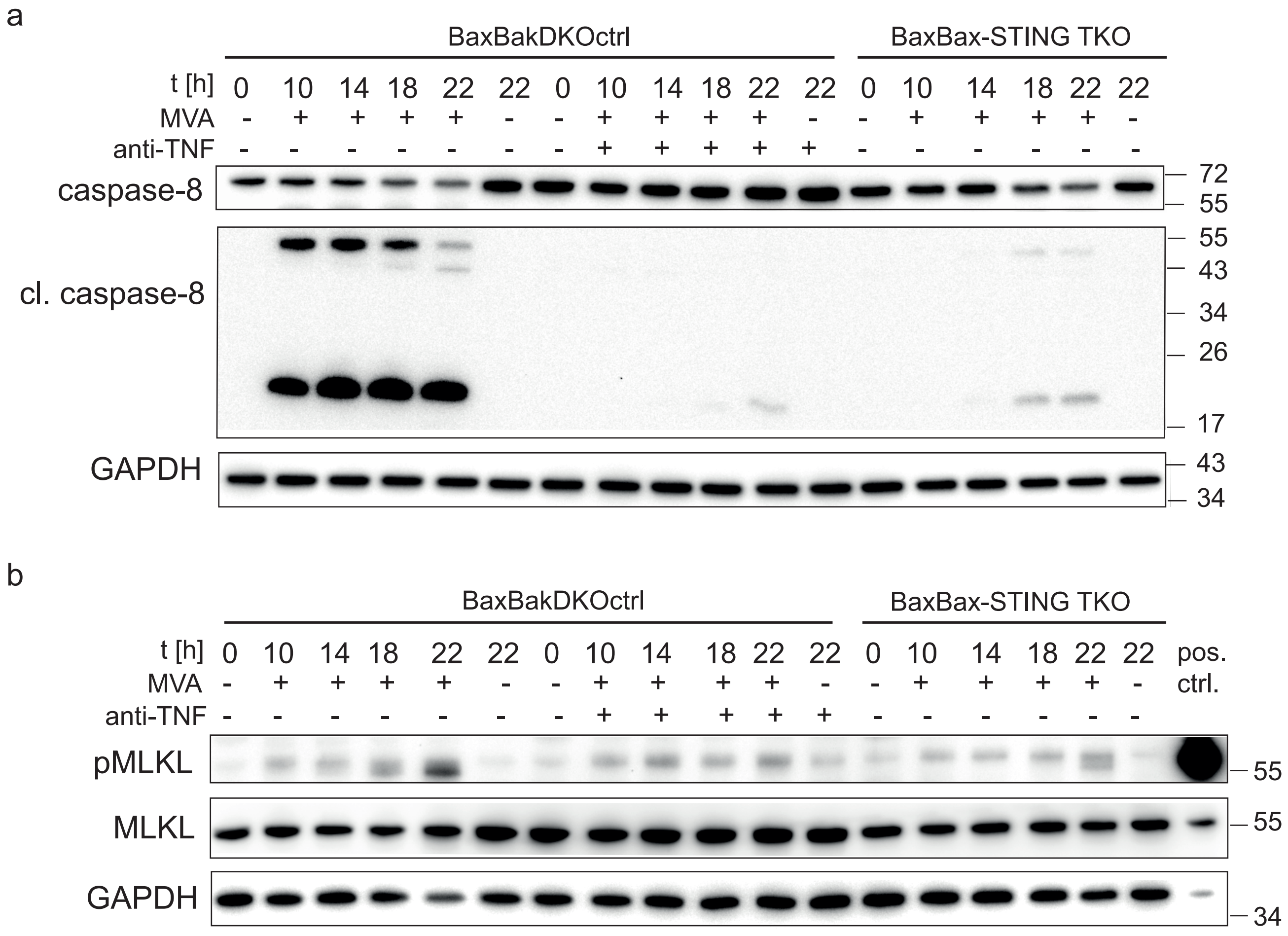
a
BaxBakDKOctrl
BaxBax-STING TKO
t [h] 0 10 14 18 22 22 0 10 14 18 22 22 0 10 14 18 22 22
MVA - + + + + - - + + + + - - + + + + -
anti-TNF - - - - - - - + + + + + - - - - - -
caspase-8
cl. caspase-8
GAPDH
72
55
55
43
34
26
17
43
34
b
BaxBakDKOctrl
BaxBax-STING TKO
t [h] 0 10 14 18 22 22 0 10 14 18 22 22 0 10 14 18 22 22 pos. ctrl.
MVA - + + + + - - + + + + - - + + + + -
anti-TNF - - - - - - - + + + + + - - - - - -
pMLKL
MLKL
GAPDH
55
55
34

Figure S11

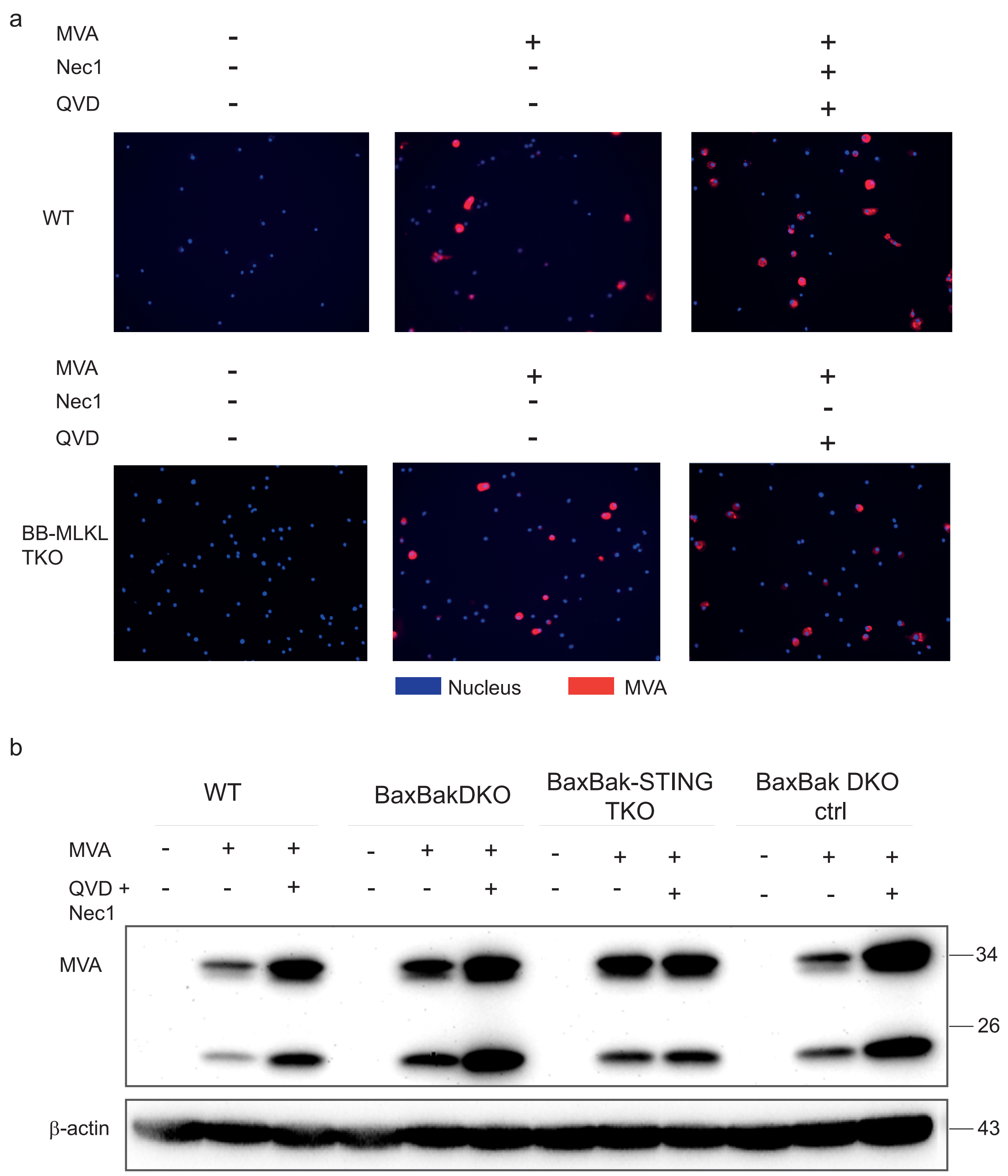

a
MVA - + +
Nec1 - - +
QVD - - +
WT
MVA - + +
Nec1 - - -
QVD - - +
BB-MLKL TKO
Nucleus
MVA
b
WT
BaxBakDKO
BaxBak-STING TKO
BaxBak DKO ctrl
MVA - + + - + + - + + - + +
QVD + Nec1 - - + - - + - - + - - +
MVA
β-actin
34
26
43

Figure S12

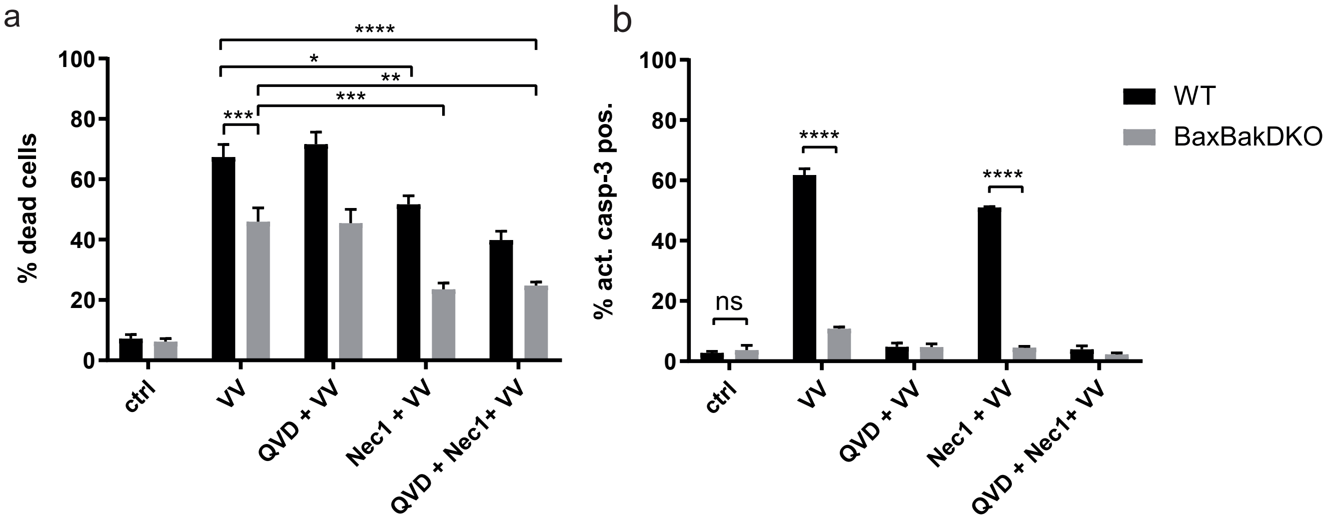
a
% dead cells
100
80
60
40
20
0
****
*
**
***
***
ctrl
VV
QVD + VV
Nec1 + VV
QVD + Nec1+ VV
b
% act. casp-3 pos.
100
80
60
40
20
0
ns
****
****
ctrl
VV
QVD + VV
Nec1 + VV
QVD + Nec1+ VV
WT
BaxBakDKO
